# Plant growth promoting characteristics of halophilic and halotolerant bacteria isolated from coastal regions of Saurashtra Gujarat

**DOI:** 10.1038/s41598-022-08151-x

**Published:** 2022-03-18

**Authors:** Likhindra Reang, Shraddha Bhatt, Rukam Singh Tomar, Kavita Joshi, Shital Padhiyar, U. M. Vyas, Jasmin Kumar Kheni

**Affiliations:** 1 Department of Biotechnology, Junagadh Agricultural University, Junagadh, Gujarat India; 2 Main Oilseed Research Station, Junagadh Agricultural University, Junagadh, Gujarat India

**Keywords:** Biotechnology, Microbiology, Plant sciences

## Abstract

Halophiles are a class of microorganisms that thrive in environments with very high salt concentrations. The coastal regions of Saurashtra Gujarat host a diverse group of microorganisms including halophilic and halotolerant bacteria that may have plant growth promoting characteristics. Microorganisms with plant growth promoting characteristics are of immense importance in the field of agriculture and the present study was conducted to investigate the characteristics of halophilic and halotolerant bacteria isolated from agricultural soils of coastal regions of Junagadh and Porbandar districts of Saurashtra, Gujarat. A total of 15 isolated strains showed indole acetic acid production, solubilization of phosphate and potash, and nitrogen fixing capacity ranging from 18.77–33.48 μg ml^−1^, 50.10–106.10%, 180.42–239.92% and 0.170–0.480 g kg^−1^ of Jensen’s agar medium, respectively, while two isolates were also found positive for siderophore production. Besides, nine out of fifteen isolates also showed positive ACC deaminase activity ranging from 0.92-5.78 µM α-ketobutyrate mg^−1^ h^−1^. The isolates were further characterized by physiological, microscopic, and biochemical tests. The halophilic and halotolerant bacterial isolates were identified by 16S rRNA gene sequencing as belonging to *Halomonas pacifica, H. stenophila*, and *Bacillus haynesii, B. licheniformis* and *Oceanobacillus aidingensis* respectively. The 16S rRNA partial gene sequence of two isolates belonging to *H. pacifica* and *H. stenophila* were submitted to NCBI with accession number MK955347 and MK961217 respectively. The findings of the present investigation showed that isolated bacterial halophiles possess promising plant growth promoting characteristics. Their potential as bioinoculants to alleviate salinity stress in crops and for bioremediation deserves further investigation.

## Introduction

Halophiles are organisms that thrive in high salt environments by balancing the osmotic pressure of the environment in order to resist the denaturing effects of salts. Most halophiles have been placed in the Archaea domain, but there are also halophiles belonging to Bacteria and Eukarya domains, for instance alga *Dunaliella salina* or fungus *Wallemia ichthyophaga*. Thus, halophiles include all the three domains of life, viz*.,* Archaea, Bacteria, and Eukarya and contain representatives of many different physiological types adapted to a wide range of salt concentrations^1^.

Halophiles have been classified by many scientists using different standards. The classification proposed by Kushner and Kamekura^2^ that groups halophiles according to their ability to survive at varying NaCl concentrations is reported to be the most widely accepted by most scientists. Based on this classification, halophilic microorganisms are divided into different categories as follows: Extreme halophiles, able to grow optimally in media with 15–30% w/v (2.5–5.2 M) NaCl. Borderline extreme halophiles, requiring at least 12% w/v NaCl. Moderate halophiles, growing optimally in media with 3–15% w/v (0.5–2.5 M) NaCl, and Slight halophiles, able to grow optimally at between 1 and 3% w/v (0.2–0.5 M) NaCl.

In contrast, non-halophilic microorganisms refer to those organisms that show optimum growth in media containing NaCl concentration less than 1% (0.2 M). However, those bacteria capable of growing irrespective of presence or absence of salt and capable of tolerating relatively high NaCl concentrations have been categorized as halotolerant or extremely tolerant if tolerance level exceeds 15% (2.5 M) NaCl. Thus, accordingly there are several categories of halotolerant microbes: Non-tolerant, those which tolerate only a small concentration of salt (about 1% w/v). Slightly tolerant, tolerating up to 6–8% (w/v). Moderately tolerant, up to 18–20% (w/v). Extremely tolerant, those microbes that grow over the whole range of salt concentrations from zero up to saturation^3^.

A considerable number of halophilic and halotolerant bacteria have been reported to possess plant growth promoting characteristics. Ramadoss et al*.*^4^ showed that halotolerant bacteria isolated from saline habitats ameliorated the salt stress in wheat seedlings. Anbumalar et al*.*^5^ investigated the plant growth promoting potentialities of IAA and phosphate solubilizing halobacterium strains and showed their effectiveness in plant seed germination and growth in various crops like cotton, tomato, lady’s finger and maize under in vitro condition. Marakana et al*.*^6^ also reported that plant growth promoting halotolerant bacteria serve as a potential tool for alleviating salinity stress in salt sensitive crops.

Saurashtra, also known as Sorath or Kathiawar, is a peninsular region of Gujarat, with natural saline habitat in western India, located on the Arabian Sea. The coastal areas of the region covering the entire sampling sites of the present investigation represent one of the natural saline microbial biodiversity hotspots in the country harboring a diverse group of microorganisms including halophilic and halotolerant bacteria that may have plant growth promoting characteristics. Previous reports on successful isolation and characterization of rhizospheric halotolerant bacteria from Bhavnagar district of Saurashtra also showed plant growth promoting potential as reported by Gontia et al*.*^7^ and Jha et al*.*^8,9^.

Symbiotic or non-symbiotic soil microorganisms that colonize the rhizosphere, inhabit plant roots and exert a positive effect to plants directly or indirectly. The use of microorganisms with the aim of improving nutrients availability for plants is an important practice and necessary for agriculture irrespective of their source of habitations throughout the world. Direct promotion of growth by PGPR occurs when the rhizobacteria produce metabolites that promote plant growth such as auxins, cytokinins, and gibberellins, ACC deaminase as well as through the solubilization of phosphate, potash, zinc minerals, nitrogen fixation etc. Indirect growth promotion occurs through the elimination of pathogens by the production of cyanide and siderophores. PGPR beneficial effects have been exploited in many areas including biofertilizers, microbial rhizoremediation and biopesticides^5^.

The present study evaluated plant growth promoting characteristics of halophilic and halotolerant bacteria isolated from agricultural soils of coastal regions of Saurashtra, Gujarat.

## Results

The results of preliminary soil analysis is presented in Table 1. The physico-chemical characteristics of the soil samples such as pH, E.C., organic carbon content, and available phosphorous and potash ranged from 7.4–8.1, 0.76–1.59 dS m^−1^, 4.03–7.47 g kg^−1^, 29.57–54.33 and 166.70–248.33 kg ha^−1^ respectively.

**Table 1:** Physico-chemical characteristics of soil samples.

Sample no.	pH	EC (dS m^−1^)	OC (g kg^−1^)	Available P_2_O_5_ (kg ha^−1^)	Available K_2_O (kg ha^−1^)
1	7.830	1.051	4.889 ± 0.026	29.570 ± 0.747	166.701 ± 2.909
2	7.920	0.816	7.467 ± 0.026	34.333 ± 0.747	201.242 ± 2.909
3	7.900	1.328	5.500 ± 0.026	37.750 ± 0.747	211.366 ± 2.909
4	7.470	1.246	4.733 ± 0.026	36.077 ± 0.747	207.480 ± 2.909
5	8.100	0.791	6.400 ± 0.026	39.467 ± 0.747	209.350 ± 2.909
6	8.130	1.006	4.033 ± 0.026	45.507 ± 0.747	221.715 ± 2.909
7	8.150	1.131	6.733 ± 0.026	49.470 ± 0.747	233.363 ± 2.909
8	8.027	1.092	6.200 ± 0.026	45.814 ± 0.747	223.393 ± 2.909
9	8.040	1.108	6.800 ± 0.026	49.140 ± 0.747	232.512 ± 2.909
10	7.960	1.573	5.500 ± 0.026	48.530 ± 0.747	231.302 ± 2.909
11	7.910	0.763	6.933 ± 0.026	49.017 ± 0.747	235.648 ± 2.909
12	7.890	1.593	4.500 ± 0.026	51.513 ± 0.747	247.251 ± 2.909
13	8.060	1.164	5.033 ± 0.026	54.333 ± 0.747	248.326 ± 2.909
14	7.979	1.138	4.733 ± 0.026	51.763 ± 0.747	233.883 ± 2.909
15	8.100	0.770	4.667 ± 0.026	53.647 ± 0.747	247.744 ± 2.909
S.Em. ±			0.018	0.528	2.057
C.D. at 5%			0.052	1.526	5.941
C.V. %			5.604	2.031	1.595

*OC* organic carbon; Values of OC, P_2_O_5_ and K_2_O are mean ± standard error of three replicates.

### Morphological characterization

The morphological characteristics of isolates deduced from colony and microscopic features are shown in Table 2. The colony morphologies of isolates ranged from irregular to circular in shape with a majority being small to medium in size with flat to raised and convex type of elevations, while some of them were pigment producing. Colony pigmentation among isolates included pale yellow, creamy, shiny, watery and pure white. Most of the colonies had entire to irregular margins, while a few had undulate margins. The isolates also displayed different cell sizes and morphologies when viewed under the microscope. The cells ranged from coccus to short or thin long rod shape in single or pairs to bunchy type organization, while few were also found to be filamentous in structure. Nine isolates were gram negative while the remaining six were gram positive. All isolates were motile and some were spore forming in nature.

**Table 2:** Colony and microscopic characteristics of halophilic bacterial isolates.

Isolates	Colony characteristics						Microscopic characteristics						
	Shape	Margin	Elevation	Texture	Opacity	Pigment	Shape	Arrangement	Gram’s reaction	Spore formation	Motility	Length (μm)	Width (μm)
S_1_	Circular	Entire	Flat	Dry	Opaque	White	Rod	Single, bunch	−ve	−ve	+ve	1.40–2.46	0.75–0.77
S_2_	Irregular	Irregular	Raised	Mucoid	Transparent	Watery shiny	Rod	Single, pair, bunch	−ve	−ve	+ve	1.61–6.78	0.71–0.83
S_3_	Circular	Entire	Flat	Mucoid	Translucent	Shiny white	Short rod	Single, pair	−ve	−ve	+ve	0.75–1.58	0.53–0.57
S_4_	Circular	Entire	Slightly raised	Dry	Opaque	Creamy white	Short rod	Single, pair	−ve	−ve	+ve	1.01–2.12	0.38 -0.40
S_5_	Irregular	Irregular	Raised	Mucoid	Opaque	Shiny white	Rod	Single, pair	−ve	−ve	+ve	0.85–1.56	0.64–0.65
S_6_	Irregular	Irregular	Slightly raised	Mucoid	Transparent	Watery shiny	Rod	Single, pair, bunch	−ve	+ve	+ve	1.32–3.20	0.55 -0.73
S_7_	Circular	Entire	Flat	Mucoid	Transparent	Watery shiny	Rod	Single, pair, bunch	−ve	−ve	+ve	1.22–2.56	0.76–0.80
S_8_	Irregular	Irregular	Raised	Mucoid	Translucent	Shiny white	Coccus	Single, pair, bunch	+ve	−ve	+ve	0.90–2.04	0.47–0.56
S_9_	Irregular	Irregular	Flat	Mucoid	Opaque	Light yellow	Rod	Single, pair	−ve	+ve	+ve	1.09–1.36	0.56–0.61
S_10_	Irregular	Irregular	Slightly raised	Mucoid	Translucent	Shiny white	Short rod	Single, pair	+ve	−ve	+ve	1.97–2.47	0.51–0.52
S_11_	Circular	Entire	Convex	Mucoid	Opaque	Creamy white	Rod	Pair, bunch	−ve	+ve	+ve	1.37–2.72	0.54–0.56
S_12_	Irregular	Irregular	Slightly raised	Mucoid	Opaque	Creamy white	Thin rod	Single, pair, bunch	+ve	−ve	+ve	1.37–2.23	0.55–0.80
S_13_	Circular	Entire	Raised	Mucoid	Transparent	Watery shiny	Short thin rod	Single, pair, bunch	+ve	−ve	+ve	1.25–2.34	0.72–0.74
S_14_	Circular	Entire	Raised	Mucoid	Opaque	Creamy white	Rod	Single, pair, bunch	+ve	−ve	+ve	0.90–1.86	0.62–0.64
S_15_	Circular	Entire	Raised	Mucoid	Opaque	Creamy white	Rod	Single, pair, bunch	+ve	−ve	+ve	1.77–3.36	0.67–0.65

### Determination of salt, pH and temperature tolerance test

The results of tolerance to different NaCl, pH and temperatures by isolates are presented in Table 3. The isolates were capable of tolerating salt concentrations up to 25% with optimum growth between 10–15% NaCl, optimum pH ranged between 6–8 with tolerance towards extreme acidity and increased growth towards alkalinity, and temperature tolerance up to 45 °C with optimum temperature observed at 35 °C.

**Table 3:** Determination of NaCl, pH and temperature tolerance test of isolates.

Isolates	NaCl tolerance (%)					pH tolerance					Temperature tolerance (°C)			
	5	10	15	20	25	2	4	6	8	10	18	25	35	45
S_1_	+++	+++	+++	+	−	+++	+++	+++	+++	++	++	++	+++	+++
S_2_	+++	++	++	+	+	−	−	+	+	−	+	+	+	+
S_3_	+++	+++	+++	++	+	−	−	+++	+++	+++	++	+++	+++	+
S_4_	+++	++	++	+	+	+++	+++	+++	+++	+++	++	+++	+++	+
S_5_	+++	+++	+++	++	−	−	−	+	++	+++	+	++	+++	+++
S_6_	+++	+++	+	+	+	−	−	+	+	++	+	+	+	+++
S_7_	+++	+++	++	+	+	−	−	+	+	−	−	+	++	++
S_8_	+++	+++	++	+	−	+	+	+	−	−	+	++	+++	+
S_9_	+++	+++	++	+	+	+	+	+	−	−	+	+	+++	+++
S_10_	+++	+++	++	+	+	−	−	+++	+	+	+	++	+++	++
S_11_	+++	+++	+	+	−	+	+	++	+	−	+	+	+++	++
S_12_	+++	++	++	++	+	−	−	+	+	++	+	+	+	−
S_13_	+++	+++	+	+	+	+	+	+	−	−	+	−	+	+
S_14_	+++	+++	+	+	+	+++	+	+	−	−	+	+	+	+
S_15_	+++	+++	+	+	+	+	+	+	+	++	+	+	++	+

‘−’: No growth; ʻ+ʼ: Minimal growth; ʻ++ʼ: Moderate growth; ʻ+++ʼ: Excellent growth.

### Biochemical characterization

The results pertaining to various biochemical tests conducted are shown in Table 4. The biochemical tests were carried out in order to determine the different biochemical characteristics of the isolates as addressed in the discussion section.

**Table 4:** Biochemical tests of isolates.

Isolates	S_1_	S_2_	S_3_	S_4_	S_5_	S_6_	S_7_	S_8_	S_9_	S_10_	S_11_	S_12_	S_13_	S_14_	S_15_
Starch hydrolysis	+ve	+ve	+ve	+ve	+ve	+ve	+ve	+ve	+ve	+ve	+ve	+ve	+ve	+ve	+ve
Lipid hydrolysis	+ve	+ve	+ve	+ve	+ve	+ve	+ve	+ve	+ve	+ve	+ve	+ve	+ve	+ve	+ve
Gelatin hydrolysis	+ve	+ve	+ve	+ve	+ve	+ve	+ve	+ve	+ve	+ve	+ve	+ve	+ve	+ve	+ve
Indole production	−ve	−ve	−ve	−ve	−ve	−ve	−ve	+ve	+ve	−ve	−ve	−ve	+ve	+ve	+ve
Methyl Red	+ve	+ve	+ve	+ve	+ve	+ve	+ve	+ve	+ve	+ve	+ve	+ve	+ve	+ve	+ve
Voges–Proskauer	−ve	−ve	−ve	−ve	−ve	−ve	−ve	−ve	−ve	−ve	−ve	−ve	−ve	−ve	−ve
H_2_S production	−ve	−ve	−ve	−ve	−ve	−ve	−ve	−ve	−ve	−ve	−ve	−ve	−ve	−ve	+ve
Catalase	+ve	+ve	+ve	−ve	+ve	+ve	−ve	+ve	+ve	+ve	+ve	−ve	+ve	+ve	+ve
Oxidase	+ve	+ve	+ve	+ve	+ve	+ve	+ve	+ve	+ve	+ve	+ve	+ve	+ve	+ve	+ve
Urease	+ve	+ve	−ve	−ve	−ve	−ve	−ve	−ve	−ve	+ve	+ve	+ve	−ve	−ve	+ve
Nitrate reduction	+ve	+ve	+ve	−ve	−ve	−ve	+ve	+ve	+ve	−ve	+ve	+ve	+ve	−ve	+ve
Phenylalanine deamination	−ve	−ve	−ve	−ve	−ve	−ve	−ve	−ve	−ve	−ve	−ve	−ve	−ve	−ve	+ve
Glucose utilization	+ve	+ve	+ve	+ve	+ve	+ve	+ve	+ve	+ve	+ve	+ve	+ve	+ve	+ve	+ve
Adonitol utilization	−ve	−ve	−ve	−ve	−ve	−ve	−ve	−ve	−ve	−ve	−ve	−ve	−ve	−ve	−ve
Lactose utilization	−ve	−ve	−ve	+ve	+ve	+ve	+ve	+ve	+ve	+ve	+ve	−ve	+ve	+ve	+ve
Arabinose utilization	+ve	−ve	+ve	+ve	+ve	+ve	+ve	+ve	+ve	+ve	+ve	+ve	+ve	+ve	+ve
Sorbitol utilization	+ve	−ve	−ve	−ve	−ve	−ve	−ve	+ve	+ve	+ve	+ve	−ve	+ve	+ve	+ve
Citrate utilization	+ve	−ve	−ve	−ve	−ve	−ve	−ve	−ve	−ve	−ve	+ve	−ve	−ve	−ve	−ve
Lysine utilization	+ve	−ve	−ve	−ve	−ve	−ve	+ve	+ve	+ve	+ve	−ve	+ve	+ve	+ve	+ve
Ornithine utilization	+ve	−ve	−ve	−ve	−ve	−ve	−ve	−ve	−ve	−ve	−ve	+ve	+ve	+ve	−ve

### IAA production

**Figure 1:**
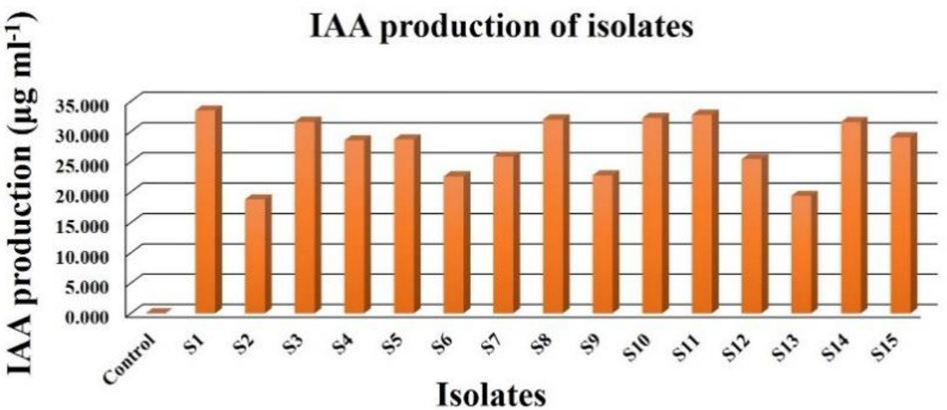
IAA production profile of isolates.

The results of indole acetic acid (IAA) production by isolates are shown in Table 5. The amount of IAA production by isolates ranged from 18.77 to 33.48 μg ml^−1^ (Fig. 1) shown by isolates S_2_ and S_1_ respectively.

**Table 5:** Indole acetic acid production and nitrogen fixing capacity of isolates.

Sr. no.	Isolate code	IAA production (μg ml^−1^) ± S.E	Nitrogen fixation capacity (g kg^−1^) ± S.E
1	S_1_	33.479 ± 0.37	0.200 ± 0.001
2	S_2_	18.769 ± 0.37	0.290 ± 0.001
3	S_3_	31.621 ± 0.37	0.240 ± 0.001
4	S_4_	28.521 ± 0.37	0.480 ± 0.001
5	S_5_	28.689 ± 0.37	0.240 ± 0.001
6	S_6_	22.564 ± 0.37	0.230 ± 0.001
7	S_7_	25.847 ± 0.37	0.190 ± 0.001
8	S_8_	31.993 ± 0.37	0.240 ± 0.001
9	S_9_	22.746 ± 0.37	0.420 ± 0.001
10	S_10_	32.235 ± 0.37	0.270 ± 0.001
11	S_11_	32.776 ± 0.37	0.170 ± 0.001
12	S_12_	25.436 ± 0.37	0.330 ± 0.001
13	S_13_	19.362 ± 0.37	0.320 ± 0.001
14	S_14_	31.583 ± 0.37	0.370 ± 0.001
15	S_15_	29.050 ± 0.37	0.340 ± 0.001
16	Control	0.000	0.000
S.Em. ±		0.264	0.001
C.D. at 5%		0.763	0.002
C.V. %		1.780	4.358
Values are mean ± standard error of three replicates

### Nitrogen fixation capacity

**Figure 2:**
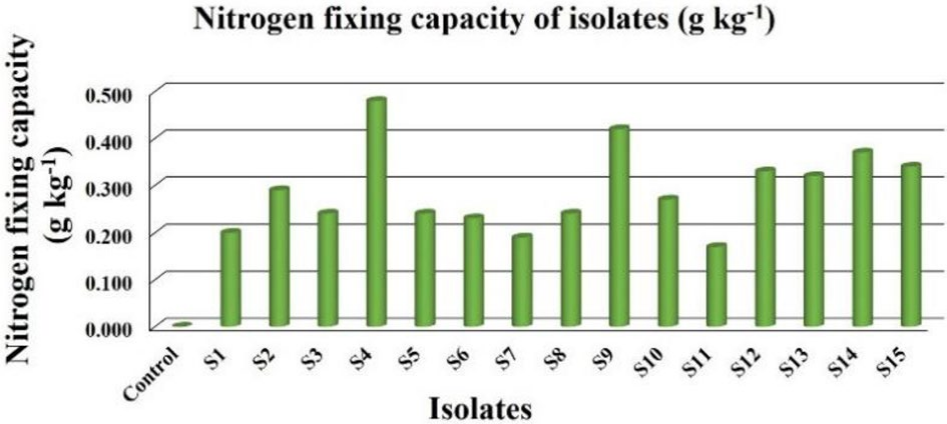
Nitrogen fixing capacity of isolates on Jensen’s agar medium.

The results of nitrogen fixing capacity of isolates are presented in Table 5 which were found to range from 0.170 to 0.480 g kg^−1^ of Jensen’s agar medium shown by isolate S_11_ and S_4_ respectively (Fig. 2). The statistical value of calculated F was greater than that of table F at both 1 and 5% respectively thus indicating a high level of significance at both 1 and 5%.

### Qualitative phosphate solubilization test in solid medium

**Figure 3:**
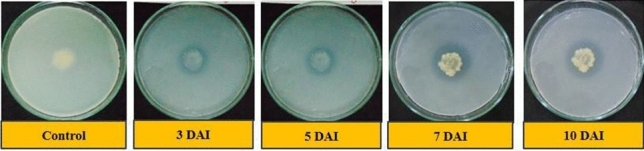
Solubilization of phosphate by isolate S_8_ on 3, 5, 7 and 10 DAI respectively.

**Figure 4:**
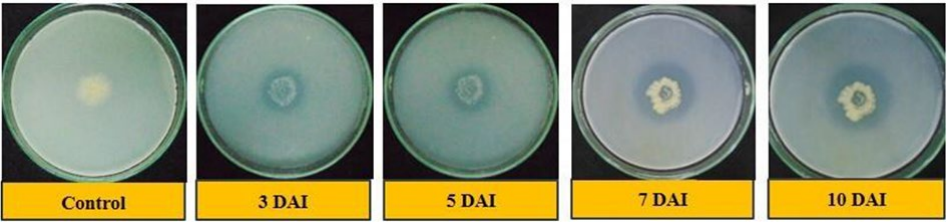
Solubilization of phosphate by isolate S_10_ on 3, 5, 7 and 10 DAI respectively.

The results observed in qualitative phosphate solubilization capacity of isolates are presented in Table 6. Thirteen out of fifteen isolates showed positive test while two (S_5_ and S_9_) isolates were negative and did not show any zone of solubilization (ZOS) on the Sperber’s agar media. The highest ZOS and thereby solubilization efficiency was shown by isolate S_8_ with ZOS of 10.40 and 13.30 mm (Fig. 3), phosphate solubilization efficiency (PSE) of 183.60 and 111.10% on 3 and 5 DAI respectively and S_10_ with ZOS of 16.30 mm (Fig. 4), PSE of 106.10% on 7 DAI. However, on 10 DAI there was very little or no increase in the ZOS in all isolates. Here, *E. coli* was kept as the negative control which showed no zone of clearance even after 10 days of inoculation on tricalcium phosphate (TCP) medium.

**Table 6:** Phosphate Solubilization Efficiency (PSE) of isolates on TCP media during 3, 5, 7 and 10 DAI.

Sr. no.	Isolates	ZOS on 3 DAI			ZOS on 5 DAI			ZOS on 7 DAI			ZOS on 10 DAI		
		ZOS (mm)	PSE (%)	S.I	ZOS (mm)	PSE (%)	S.I	ZOS (mm)	PSE (%)	S.I	ZOS (mm)	PSE (%)	S.I
1	S_1_	6.70	71.90 ± 3.23^(h)^	1.70	10.50	67.40 ± 1.88^(ef)^	1.70	12.60	73.30 ± 1.87^(e)^	1.70	12.60	73.60 ± 1.87^(e)^	1.70
2	S_2_	3.60	41.80 ± 3.23^(i)^	1.40	7.90	47.40 ± 1.88^(h)^	1.50	11.80	68.00 ± 1.87^(f)^	1.70	11.80	68.00 ± 1.87^(f)^	1.70
3	S_3_	5.50	105.20 ± 3.23^(f)^	2.10	11.30	77.90 ± 1.88^(d)^	1.80	14.40	77.50 ± 1.87^(de)^	1.80	14.40	77.50 ± 1.87^(de)^	1.80
4	S_4_	7.70	141.30 ± 3.23^(b)^	2.40	11.60	80.30 ± 1.88^(cd)^	1.80	14.70	90.80 ± 1.87^(c)^	1.90	16.00	94.90 ± 1.87^(c)^	1.90
5	S_6_	6.00	96.80 ± 3.23^(fg)^	2.00	9.60	54.50 ± 1.88^(g)^	1.50	9.60	55.70 ± 1.87^(g)^	1.60	9.70	55.70 ± 1.87^(g)^	1.60
6	S_7_	6.80	93.70 ± 3.23^(g)^	1.90	10.80	69.60 ± 1.88^(e)^	1.70	9.80	60.30 ± 1.87^(g)^	1.60	9.80	60.30 ± 1.87^(g)^	1.60
7	S_8_	10.40	183.60 ± 3.23^(a)^	2.80	13.30	111.10 ± 1.88^(a)^	2.10	12.30	74.30 ± 1.87^(e)^	1.70	12.60	74.30 ± 1.87^(e)^	1.70
8	S_10_	7.70	120.50 ± 3.23^(c)^	2.20	11.60	81.90 ± 1.88^(cd)^	1.80	16.30	106.10 ± 1.87^(a)^	2.10	16.30	106.10 ± 1.87^(a)^	2.10
9	S_11_	9.10	144.00 ± 3.23^(b)^	2.40	12.90	100.50 ± 1.88^(b)^	2.00	15.10	97.00 ± 1.87^(b)^	2.00	15.10	97.00 ± 1.87^(b)^	2.00
10	S_12_	4.50	80.40 ± 3.23^(h)^	1.80	7.20	47.50 ± 1.88^(h)^	1.50	8.30	50.10 ± 1.87^(h)^	1.50	8.30	50.10 ± 1.87^(h)^	1.50
11	S_13_	7.30	106.40 ± 3.23^(e)^	2.10	11.30	84.50 ± 1.88^(c)^	1.80	13.90	81.30 ± 1.87^(d)^	1.80	13.90	81.30 ± 1.87^(d)^	1.80
12	S_14_	5.60	106.50 ± 3.23^(d)^	2.10	8.80	56.40 ± 1.88^(g)^	1.60	8.50	50.50 ± 1.87^(h)^	1.50	8.50	50.50 ± 1.87^(h)^	1.50
13	S_15_	6.30	96.00 ± 3.23^(fg)^	2.00	9.60	62.80 ± 1.88^(f)^	1.60	12.60	73.80 ± 1.87^(e)^	1.70	12.60	73.80 ± 1.87^(e)^	1.70
14	Control	0.00	0.00	0.00	0.00	0.00	0.00	0.00	0.00	0.00	0.00	0.00	0.00
S.Em. ±		2.28			1.33			1.32			1.32		
C.D. at 5%		6.64			3.86			3.84			3.84		
C.V. %		3.70			3.17			3.10			3.09		

*PSE* phosphate solubilization efficiency, *ZOS* zone of solubilization, *S.I*. solubilization index. Values of PSE (%) are mean ± standard error of 3 replicates. Different letters at various points indicate significant difference between treatments using Duncan’s multiple range test (P ≤ 0.01).

### Quantitative phosphate solubilization test in liquid medium

The quantitative phosphate solubilization capacity of isolates were estimated by measuring the drop in pH and release of Pi (inorganic phosphorous) in TCP broth recorded at intervals of 3, 5, 7 and 10 DAI (Table 7). The drop in broth pH of respective isolates were found to increase with incubation time for all the isolates and the maximum drop in pH (4.1 ± 0.09) was recorded in broth of isolate S_8_ at 10 DAI followed by isolates S_11_, S_4_ and S_10_ with drop in pH of (4.3 ± 0.09), (4.4 ± 0.09), (4.5 ± 0.09), respectively. The amount of Pi released from TCP also increased with incubation time for all isolates and the maximum Pi released (728.83 ± 16.59 μg ml^−1^) followed by (715.50 ± 16.59 μg ml^−1^), and (708.60 ± 16.59 μg ml^−1^) were recorded in isolate S_11_ followed by isolates S_4_, and S_8_ etc. respectively. The uninoculated control tube showed a drop in pH up to (6.5 ± 0.09) at 10 DAI with very little soluble Pi released. So, there was a negative correlation between pH drop and Pi release in TCP broth (Table 8). The statistical evaluation of C.V. per cent of Pi released (μg ml^−1^) and drop in pH in TCP broth of respective isolates indicated a high level of significance with greater value of calculated F than that of table F at both 1 and 5% respectively.

**Table 7:** pH drop and Pi released from TCP broth by isolates on 3, 5, 7 and 10 DAI.

Isolate code	3 DAI		5 DAI		7 DAI		10 DAI	
	pH ± SE	Pi (μg/ml) ± SE	pH ± SE	Pi (μg/ml) ± SE	pH ± SE	Pi (μg/ml) ± SE	pH ± SE	Pi (μg/ml) ± SE
S_1_	5.6 ± 0.07^(g)^	401.69 ± 5.41^(i)^	5.4 ± 0.08^(g)^	479.79 ± 7.13^(ghi)^	5.2 ± 0.08^(ef)^	550.98 ± 10.18^(i)^	5.2 ± 0.09^(hi)^	593.12 ± 16.59^(fghi)^
S_2_	5.5 ± 0.07^(f)^	393.83 ± 5.41^(i)^	5.2 ± 0.08^(e)^	472.17 ± 7.13^(ghi)^	5.1 ± 0.08^(d)^	565.74 ± 10.18^(ghi)^	4.9 ± 0.09^(ef)^	578.60 ± 16.59^(fghi)^
S_3_	5.7 ± 0.07^(h)^	466.21 ± 5.41^(f)^	5.6 ± 0.08^(h)^	536.69 ± 7.13^(e)^	5.4 ± 0.08^(gh)^	615.98 ± 10.18^(def)^	5.2 ± 0.09^(ghi)^	657.64 ± 16.59^(cde)^
S_4_	5.0 ± 0.07^(c)^	543.36 ± 5.41^(c)^	4.8 ± 0.08^(c)^	622.88 ± 7.13^(b)^	4.6 ± 0.08^(b)^	700.98 ± 10.18^(c)^	4.4 ± 0.09^(c)^	715.50 ± 16.59^(ab)^
S_6_	5.9 ± 0.07^(i)^	458.60 ± 5.41^(fg)^	5.6 ± 0.08^(h)^	522.64 ± 7.13^(ef)^	5.4 ± 0.08^(h)^	593.12 ± 10.18^(fg)^	5.3 ± 0.09^(hi)^	610.02 ± 16.59^(f)^
S_7_	5.7 ± 0.07^(h)^	422.17 ± 5.41^(h)^	5.6 ± 0.08^(h)^	486.45 ± 7.13^(gh)^	5.3 ± 0.08^(fg)^	572.17 ± 10.18^(ghi)^	5.1 ± 0.09^(gh)^	601.45 ± 16.59^(fgh)^
S_8_	4.6 ± 0.07^(a)^	623.83 ± 5.41^(a)^	4.4 ± 0.08^(a)^	671.21 ± 7.13^(a)^	4.3 ± 0.08^(a)^	695.50 ± 10.18^(ab)^	4.1 ± 0.09^(a)^	708.60 ± 16.59^(ab)^
S_10_	5.1 ± 0.07^(d)^	509.07 ± 5.41^(d)^	5.0 ± 0.08^(d)^	543.83 ± 7.13^(d)^	4.7 ± 0.08^(c)^	664.79 ± 10.18^(c)^	4.5 ± 0.09^(d)^	685.74 ± 16.59^(abc)^
S_11_	4.9 ± 0.07^(b)^	600.98 ± 5.41^(b)^	4.7 ± 0.08^(b)^	622.17 ± 7.13^(b)^	4.5 ± 0.08^(b)^	700.74 ± 10.18^(ab)^	4.3 ± 0.09^(b)^	728.83 ± 16.59^(a)^
S_12_	6.5 ± 0.07^(j)^	409.07 ± 5.41^(hi)^	5.6 ± 0.08^(h)^	486.45 ± 7.13^(g)^	5.4 ± 0.08^(h)^	574.07 ± 10.18^(ghi)^	5.3 ± 0.09^(i)^	594.07 ± 16.59^(fghi)^
S_13_	5.5 ± 0.07^(f)^	475.02 ± 5.41^(e)^	5.3 ± 0.08^(f)^	550.98 ± 7.13^(d)^	5.1 ± 0.08^(d)^	627.41 ± 10.18^(de)^	5.0 ± 0.09^(ef)^	684.07 ± 16.59^(abcd)^
S_14_	5.3 ± 0.07^(e)^	484.79 ± 5.41^(e)^	5.2 ± 0.08^(e)^	565.02 ± 7.13^(c)^	5.1 ± 0.08^(de)^	637.64 ± 10.18^(cd)^	4.9 ± 0.09^(e)^	668.36 ± 16.59^(bcde)^
S_15_	5.7 ± 0.07^(h)^	444.07 ± 5.41^(g)^	5.4 ± 0.08^(g)^	507.17 ± 7.13^(f)^	5.3 ± 0.08^(fgh)^	586.93 ± 10.18^(fgh)^	5.1 ± 0.09^(efg)^	605.02 ± 16.59^(fg)^
Control	6.8 ± 0.07^(k)^	37.17 ± 5.41^(j)^	6.6 ± 0.08^(i)^	37.88 ± 7.13^(j)^	6.6 ± 0.08^(i)^	38.60 ± 10.18^(j)^	6.5 ± 0.09^(j)^	39.31 ± 16.59^(j)^
S.Em. ±	0.05	3.82	0.06	5.04	0.06	7.20	0.06	11.73
C.D. at 5%	0.14	11.07	0.16	14.61	0.17	20.85	0.19	33.98
C.V. %	1.51	1.48	1.78	1.72	1.20	2.15	2.23	3.36

*Pi* inorganic phosphorous. Values of pH and Pi released (μg ml^−1^) are mean ± standard error of 3 replicates. Different letters at various points indicate significant difference between treatments using Duncan’s multiple range test (P ≤ 0.01).

**Table 8:** Correlation data between pH change and Pi release in isolates broth.

	3 DAI		5 DAI		7 DAI		10 DAI	
	pH	Pi (μg ml^−1^)	pH	Pi (μg ml^−1^)	pH	Pi (μg ml^−1^)	pH	Pi (μg ml^−1^)
**3 DAI**								
pH	1							
Pi (μg ml^−1^)	− 0.85557**	1						
**5 DAI**								
pH	0.940964**	− 0.91915**	1					
Pi (μg ml^−1^)	− 0.8143**	0.987562**	− 0.88956**	1				
**7 DAI**								
pH	0.932149**	− 0.94309**	0.989688**	− 0.91634**	1			
Pi (μg ml^−1^)	− 0.77737**	0.969077**	− 0.85617**	0.988382**	− 0.89654**	1		
**10 DAI**								
pH	0.939179**	− 0.94093**	0.987197**	− 0.91078**	0.99507**	− 0.89243**	1	
Pi (μg ml^−1^)	− 0.76394**	0.960342**	− 0.83465**	0.983478**	− 0.8789**	0.996916**	− 0.87176**	1

**Means significant at P = 0.05 and 0.01.

### Potash solubilization capacity

**Figure 5:**
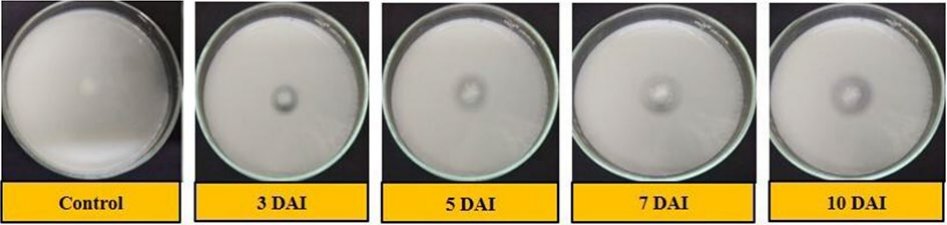
Solubilization of potash by isolate S_4_ on 3, 5, 7 and 10 DAI respectively.

The results pertaining to potash solubilization capacity of isolates are presented in Table 9. Only eight out of fifteen isolates were found positive for potash solubilization capacity in the Aleksandrow’s media. The highest ZOS and thereby solubilization efficiency was shown by isolate S_4_ with ZOS of 9.60, 12.70, 17.23 and 17.83 mm (Fig. 5), potash solubilization efficiency (KSE) of 202.97, 195.46, 241.13 and 239.92% on 3, 5, 7 and 10 DAI respectively. The *E. coli* which was included as a negative control showed no zone of solubilization even at 10 DAI. The KSE (%) was seen to increase rapidly from 3 to 7 DAI and then continued to increase slowly from 7 to 10 DAI for majority of the isolates.

**Table 9:** Potash Solubilization Efficiency (KSE) of isolates on Aleksandrow’s media during 3, 5, 7 and 10 DAI.

Sr. no.	Isolates	ZOS on 3 DAI			ZOS on 5 DAI			ZOS on 7 DAI			ZOS on 10 DAI		
		ZOS (mm)	KSE (%)	S.I.	ZOS (mm)	KSE (%)	S.I.	ZOS (mm)	KSE (%)	S.I.	ZOS (mm)	KSE (%)	S.I.
1	S_1_	5.63	109.92 ± 5.46^(cd)^	2.10	9.53	134.30 ± 5.81^(d)^	2.34	13.37	167.13 ± 5.27^(c)^	2.67	14.73	180.42 ± 1.97^(e)^	2.80
2	S_2_	6.93	118.71 ± 5.46^(bcd)^	2.19	11.20	152.50 ± 5.81^(bc)^	2.53	15.40	196.61 ± 5.27^(b)^	2.97	16.50	202.88 ± 1.97^(b)^	3.03
3	S_3_	5.23	101.99 ± 5.46^(d)^	2.02	9.37	132.97 ± 5.81^(d)^	2.33	13.70	172.84 ± 5.27^(c)^	2.73	14.87	182.05 ± 1.97^(e)^	2.82
4	S_4_	9.60	202.97 ± 5.46^(a)^	3.03	12.70	195.46 ± 5.81^(a)^	2.96	17.23	241.13 ± 5.27^(a)^	3.41	17.83	239.92 ± 1.97^(a)^	3.40
5	S_6_	6.47	128.91 ± 5.46^(b)^	2.29	10.20	165.44 ± 5.81^(bc)^	2.65	14.63	195.17 ± 5.27^(b)^	2.95	15.80	205.18 ± 1.97^(b)^	3.05
6	S_7_	6.57	123.32 ± 5.46^(bc)^	2.23	10.40	167.74 ± 5.81^(b)^	2.68	14.77	191.76 ± 5.27^(b)^	2.92	15.60	196.65 ± 1.97^(c)^	2.97
7	S_8_	6.27	120.57 ± 5.46^(bc)^	2.21	9.97	162.52 ± 5.81^(bc)^	2.63	14.13	190.99 ± 5.27^(b)^	2.91	15.53	200.86 ± 1.97^(bc)^	3.01
8	S_10_	5.83	114.42 ± 5.46^(cd)^	2.14	9.57	160.45 ± 5.81^(bc)^	2.60	13.80	185.58 ± 5.27^(b)^	2.86	14.80	188.13 ± 1.97^(d)^	2.88
9	Control	0.00	0.00	0.00	0.00	0.00	0.00	0.00	0.00	0.00	0.00	0.00	0.00
S.Em. ±		3.86			4.11			3.73			1.39		
C.D. at 5%		11.567			12.321			11.171			4.171		
C.V. %		5.237			4.479			3.350			1.208		

*KSE* Potash Solubilization Efficiency, *ZOS* Zone of Solubilization, *S.I.* Solubilization Index. Values of KSE (%) are mean ± standard error of 3 replicates. Different letters at various points indicate significant difference between treatments using Duncan’s multiple range test (P ≤ 0.01).

### Siderophore production potentiality

**Figure 6:**
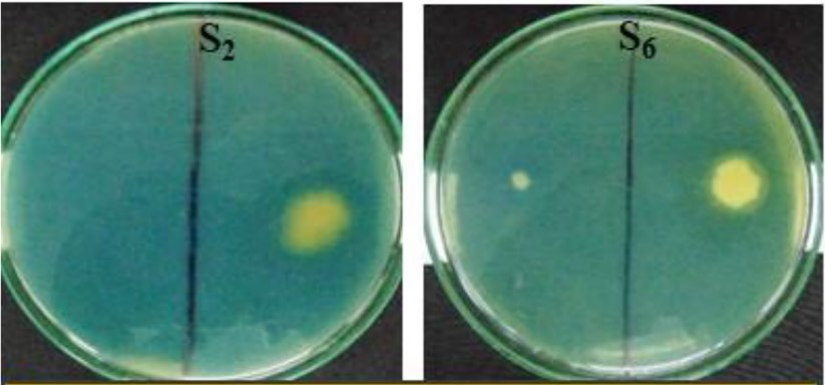
Siderophore produced by isolate S_2_ and S_6_.

**Figure 7:**
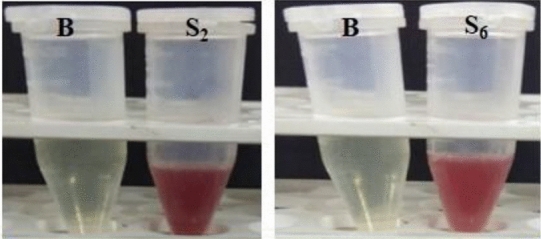
Tetrazolium test (Hydroxymate type Siderophore).

The results of siderophore production potentiality of isolates showed that only two out of fifteen isolates viz*.,* S_2_ and S_6_ were positive for siderophore production (Fig. 6). The siderophore type produced by these two isolates were further confirmed by tetrazolium test as hydroxymate type (Fig. 7) which was inferred by the instant appearance of deep red colour on addition of tetrazolium salt and NaOH to the test samples.

The results of qualitative and quantitative ACC deaminase production by isolates are shown in Table 10. Nine out of fifteen isolates were found positive for ACC deaminase activity while remaining six isolates showed negative activity. The amount of ACC deaminase production by isolates ranged from 0.92 to 5.78 μM α-ketobutyrate (α-KB) mg^−1^ h^−1^ shown by isolates S9 and S15 respectively.

**Table 10:** Qualitative and quantitative ACC deaminase production profile of isolates.

Isolate	Qualitative ACC deaminase test	Quantitative ACC deaminase production (μM α-KB mg^−1^ h^−1^ ± S.E.)
S1	+ve	3.94 ± 0.01
S2	+ve	3.46 ± 0.01
S3	+ve	1.92 ± 0.01
S4	−ve	0.00 ± 0.01
S5	−ve	0.00 ± 0.01
S6	−ve	0.00 ± 0.01
S7	−ve	0.00 ± 0.01
S8	+ve	1.24 ± 0.01
S9	+ve	0.92 ± 0.01
S10	−ve	0.00 ± 0.01
S11	+ve	4.39 ± 0.01
S12	+ve	5.16 ± 0.01
S13	+ve	3.83 ± 0.01
S14	−ve	0.00 ± 0.01
S15	+ve	5.78 ± 0.01
Control	−ve	0.00
S.Em. ±		0.01
C.D. at 5%		0.02
C.V. %		0.50

Values are mean ± standard error of three replicates.

### Molecular identification and phylogenetic analysis

**Figure 8:**
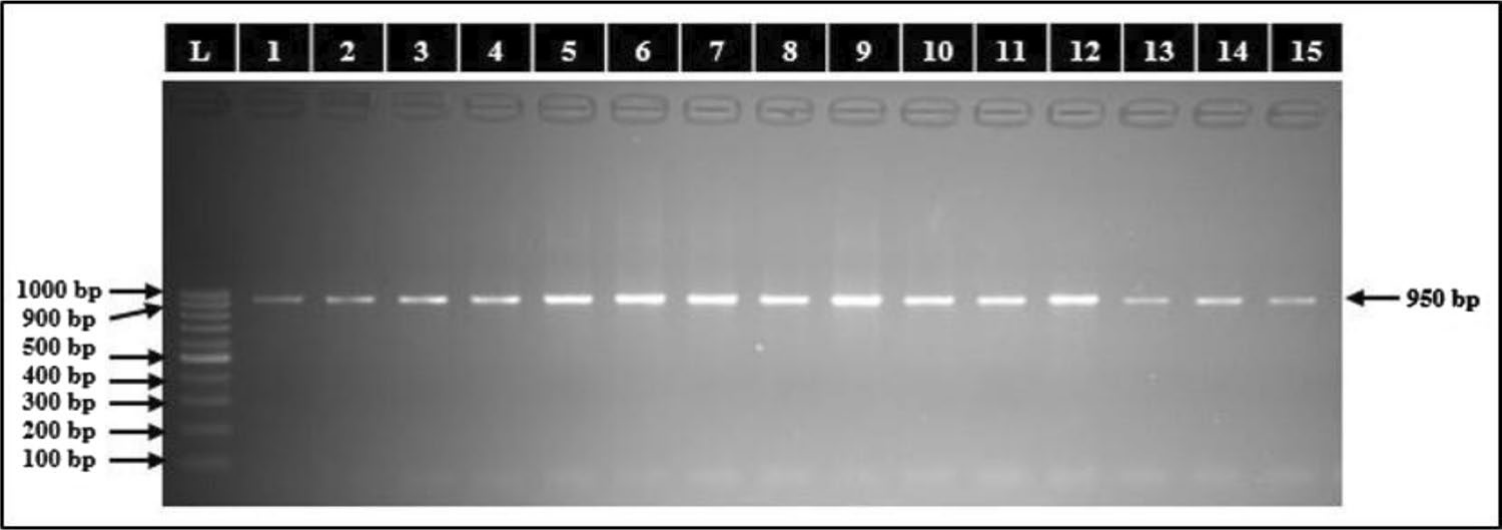
Agarose gel electrophoretic band of 1 kb ladder (Lane L) and PCR purified bands of isolates total genomic DNA extracts with size of 950 bp (Lane 1 to 5).

**Figure 9:**
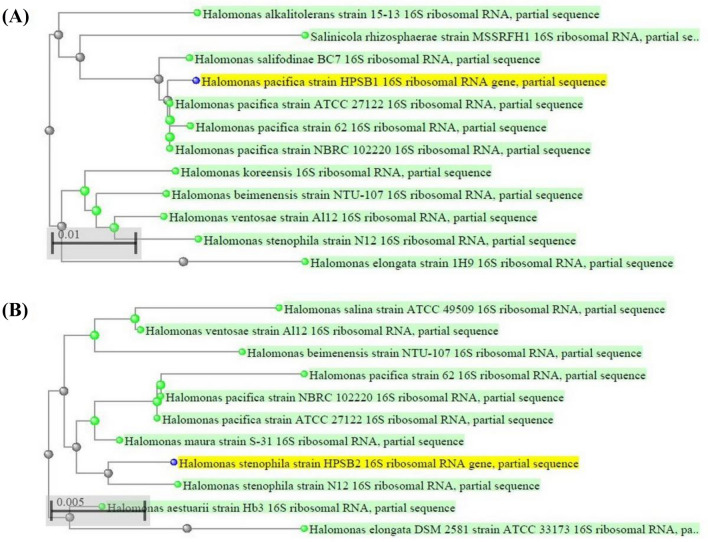
Phylogenetic dendrograms, based on partial 16S rRNA nucleotide sequences, showing the relationship between the selected halophilic bacterial strains HPSB1 and HPSB2 with closely related taxa of (A) *H. pacifica* and (B) *H. stenophila*, respectively. The blast names color map in green dots and label represents the related strains as g-proteobacteria while blue dots in yellow label represents the unknown query strains.

The halophilic bacterial isolates studied were identified by 16S rRNA gene sequencing on capillary sequencer (Applied Bio Systems 3130) using a pair of 16S universal primers. The PCR purified bands of isolates total genomic DNA extracts has been shown in (Fig. 8). The 16S rRNA gene sequences obtained were compared against the nucleotide sequences available in BLASTn Search in GenBank of NCBI. Comparative sequence analysis of BLASTn search on NCBI revealed that eight out of ten isolates designated as S_1_, S_3_, S_4_, S_5_, S_6_, S_7_, S_8_ and S_11_ belonging to genus *Halomonas* were identified as *H. pacifica* while two isolates designated as S_2_ and S_9_ were identified as *H. stenophila.* On the other hand, three out of four isolates designated as S_12_, S_14_ and S_15_ belonging to genus *Bacillus* were identified as *B. haynesii* while one isolate designated as S_13_ was identified as *B. licheniformis* and the remaining one isolate designated as S_10_ belonging to genus *Oceanobacillus* was identified as *O. aidingensis.* The partial 16S rRNA gene sequence of two isolates S_1_ and S_2_ were re-designated as *Halomonas pacifica* strain HPSB1 and *Halomonas stenophila* strain HPSB2 and submitted to NCBI with accession numbers MK955347 and MK961217 respectively. The Phylogenetic analysis of these two strains using NCBI BLAST pairwise alignments revealed their relatedness with other strains of respective species (Fig. 9) and similarly for remaining isolates (Supplementary Fig. S1 to S13).

## Discussion

Preliminary analysis showed that the soils from which the halophilic bacteria were isolated were slight to moderately alkaline, non-saline and low to medium with respect to availability of soil organic carbon, phosphorous and potash. These results agrees with the findings of Echigo et al.^10^ who reported that isolation of extreme halophiles can also occur from environments in which they are not expected to be actively growing.

The morphological characteristics of isolates deduced from the observed colony characteristics recorded in terms of size, shape, elevation, margin, texture, opacity, pigment and microscopic features showed the typical halophilic like colonies on media after 24 h of inoculation. These findings are similar to those by Kambura^11^ and Roohi et al*.*^12^.

The results of salt, pH and temperature tolerance showed that the isolates were moderate to extreme halophiles and moderate halotolerants, slightly acidophilic to alkaliphilic and mesophilic in nature with respect to their optimum growth and tolerance capacity at different levels of salt concentration, pH, and temperature.

Results from various biochemical tests conducted on isolates indicated that they were all capable of producing alpha-amylase, lipase and gelatinase enzymes, while five isolates were positive for indole production. A positive methyl red test for all isolates indicated that they are glucose fermenters. Hence, all the isolates showed negative Voges–Proskauer test as bacteria can give only either of the test positive except for some gram negative bacteria in exceptional conditions that are reported to give positive test for both methyl red and Voges–Proskauer test. On the other hand only one isolate tested positive for hydrogen sulfide production implying that only one of the fifteen isolates has an ability to reduce sulfur containing compounds to hydrogen sulfide during metabolism. All fifteen isolates tested positive for oxidase test whereas only twelve and five out of fifteen isolates were found positive for catalase and urease tests respectively. Ten out of fifteen isolates were found positive for nitrate reduction test. Only one out of fifteen isolates was detected positive for phenylalanine deamination test. All isolates were found capable of using glucose as their carbon source for energy. The same goes for arabinose except one isolate which tested negative for utilizing it as carbon source. Eleven, eight, seven, four, and two isolates tested positive in utilizing lactose, lysine, sorbitol, ornithine and citrate as carbon source respectively. However, no isolates were found positive to utilize adonitol as carbon source for their energy.

The amount of IAA produced by the isolates were found to be slightly more than the range 1 to 23 μg ml^−1^ reported by Verma et al*.*^13^ and Khamna et al*.*^14^. The results of mean comparison related to different isolates indicated that concentrations of IAA produced by the isolates under study are in agreement with the results of former studies reported by Padder et al*.*^15^ and Nghia et al*.*^16^. In their reports, the minimum to maximum IAA production at varying concentration of tryptophan in the medium ranged from 16.21 to 32.41 μg ml^−1^ and 10.44 to 33.13 μg ml^−1^ respectively. However, the IAA production range of the isolates under study are much lower as compared to the range 78 to 101 μg ml^−1^ reported by Nabti et al*.*^17^.

The positive nitrogen fixation capacity of the isolates quantified by micro Kjeldahl method even though in small quantity indicated the ability of the isolates to fix soil atmospheric nitrogen. Kanimozhi and Panneerselvam^18^ also used the similar method and quantified the total amount of nitrogen fixed by *Azospirillum* isolates in nitrogen-free medium ranging from 3.3 to 15.6 mg N g^−1^. Nitrogen fixation by free living organisms is a process that needs considerable amounts of organic matter to be effective. The free living organisms are said to be able to fix between 12 and 30 g N kg^−1^ of carbon source^19^. Thus the results of nitrogen fixation obtained from the isolates can be considered negligible to very low as compared to those of free living biological nitrogen fixers.

The solubilization of phosphates were confirmed from the formation of clear zones which is concerned with the P-solubilization by the isolates. These results implied that P-solubilization may be due to secretion of some substances into surroundings during the course of growing, which can solubilize phosphate or organophosphate. Phosphorous solubilization results are reported to vary depending on kinds of the metabolin, how quickly it is released, and also the degree of its spread on the medium. Therefore, observational method of P solubilizing zone can only be used for qualitative assays^20^. In the present study, the expansion of diameter of ZOS of the isolates during the interval of 5 to 10 DAI were found to be much lower than the range of 25 to 30 mm reported by Zhu et al*.*^20^.

The halophilic and halotolerant bacteria studied in the present investigation lowered the pH of the TCP broth. The maximum drop in pH in TCP broth recorded in isolates were in accordance with those found in halophilic bacterium strain *Kushneria *sp. YCWA18 by Zhu et al*.*^20^. However, the maximum release of soluble Pi from TCP broth in culture supernatant accompanied by the significant drop in pH i.e., acidification of TCP broth by isolates were significantly higher than that reported in the latter. This indicated the implication of organic acid production in isolates. The negative correlation between drop in pH and Pi release, on the other hand, corroborates a similar observation made even with rhizobium species^21^ and other bacteria mobilizing P from rock phosphate^22,23^. Several mechanisms like the process of acidification, chelation and exchange reaction or the production of low molecular weight organic acids have been implicated to render insoluble phosphates into soluble form^24^.

The Duncan’s Multiple Range Test (DMRT) showed varying degree of significant differences in the treatment mean of potash solubilization efficiency between the isolates tested as shown in Table 9. The maximum potash solubilization efficiency (KSE) of the isolates were found to be lower by half than that reported in potash solubilizing bacteria by Sun et al*.*^25^. However, they were within the range of 169–423% reported in the latter. Similarly, the maximum potassium solubilization index (KSI) of isolates were also slightly lower but in compliance with the range of 2.37–5.25 accounted in potash solubilizing bacteria by Fatharani and Rahayu^26^. The measure of ZOS range of isolates studied were also within the range of 20.7–28.1 mm recorded on 7 DAI in potash solubilizing bacteria by Saha et al*.*^27^. The potash solubilization capacity of isolates indicated their potential to solubilize potash in agricultural soils thus making them available to the plants. This solubilization could be attributed to excretion of organic acids which either directly dissolves rock K or chelate silicon ions to bring K into solution^28–31^. Therefore, the application of K solubilizing halophilic bacteria could be of promising approach for increasing K availability in soils cultivated for high-K-demanding crops^32–34^.

Siderophores are said to be produced especially in an environment where the iron is deficient thus causing the bacteria to excrete siderophore in the extracellular environment to sequester and solubilize iron. The inability of the isolates under study to produce siderophores can thus be attributed to the possible sufficiency of iron in their habitual environments. An investigation about connection between iron homeostasis and the osmostress response in the halophiles also reported a decrease in the requirement for both iron and histidine and a lower level of siderophore synthesis at high salinity^5^. This report also correlates with the nature of isolates in the present study being high salt requiring and hence very low to no siderophore production in such high salinity.

The amount of ACC deaminase enzyme produced by the isolates were found to be three to six folds higher than the range 325–997 nmol mg^−1^ protein h^−1^ reported in halophilic and halotolerant bacterial strains by Mukhtar et al*.*^35^ but two folds lower than 11,172.1 nM mg^−1^ protein h^−1^ reported in halotolerant bacterial isolates by Tiwari et al*.*^36^. However, the ACC deaminase production of the isolates under study are in compliance with the range 0.69–4.90 μmol α-ketobutyrate mg^−1^ protein h^−1^ reported in halotolerant bacterial strains by Siddikee et al*.*^37^. Penrose and Glick^38^ described that different organisms with a wide range of ACC deaminase activity can act as PGPR. A low level of ACC deaminase activity, approximately ≥ 20 nmol α-ketobutyrate mg^−1^ h^−1^ is sufficient to permit a bacterium to grow on ACC and to act as a PGPR. Organisms with higher levels of ACC deaminase activity that is from 300 to 400 nmol α-ketobutyrate mg^−1^ h^−1^ do not necessarily promote root elongation to any greater extent than the strains that contain less enzyme activity^38^. The positive ACC deaminase activity of the isolates thus implies their ability to irreversibly cleave ACC molecules (intermediate precursor formed by the conversion of S-Adenosyl Methionine (SAM) (itself converted from main ‘precursor of ethylene’ called methionine by SAM synthase) by ACC-synthase which is further converted by ACC-oxidase into ethylene^39^) into α-ketobutyrate and ammonia^40^, and may thereby promote plant growth by alleviating stress by lowering ethylene levels^41^ under various stress conditions.

## Methods

The study was carried out at “Department of Biotechnology, College of Agriculture, Junagadh Agricultural University, Junagadh” during the academic year 2018–2019. A total of fifteen isolates was used for studying the various plant growth promoting activities with three replication each for each experiment using a completely randomized design (CRD).

### Soil sample collection

The soil samples were collected from coastal regions of Saurashtra, Gujarat viz*.,* Junagadh and Porbandar districts located at coordinates 21.52°N 70.47°E and 21°37′48″N 69°36′0″E respectively. The names of the sites within the respective districts from which the soil samples were collected with due consultation and permissions have been shown in Table 11. A total of 15 soil samples (approximately 100 g each) were collected from agricultural fields from a depth of 5 cm around the crop rhizospheres with the help of an agricultural soil sampler.

**Table 11:** Details of soil sampling sites and locations.

Sr. no.	Sampling site	Crop rhizosphere	District	Sr. no.	Sampling site	Crop rhizosphere	District
1	Mangrol	Chilli	Junagadh	9	Madhavpur Ghed	Soyabean	Porbandar
2	Kankana	Cluster bean	Junagadh	10	Gorsar	Pigeon pea	Porbandar
3	Chankhva	Sorghum	Junagadh	11	Untada	Okra	Porbandar
4	Mekhdi	Maize	Junagadh	12	Navi Bandar	Cotton	Porbandar
5	Kalej	Indian bean	Junagadh	13	Tukada	Groundnut	Porbandar
6	Azak	Cowpea	Junagadh	14	Odadar	Brinjal	Porbandar
7	Divasa	Spine gourd	Junagadh	15	Porbandar	Black gram	Porbandar
8	Shil	Smooth gourd	Junagadh	–	–	–	–

### Preliminary soil analysis

The preliminary analysis of soil samples were carried out to determine the soil chemical properties viz*.,* soil pH by potentiometry and electrical conductivity by conductometry method^42^, and soil organic carbon content by back titration method^43^, available soil phosphorous by colorimetric method^44^ and potash by flame photometry method^42^.

### Isolation of halophilic bacteria from soil samples

Halophilic bacteria were isolated from 15 different soil samples and inoculated by streak plate method on freshly prepared halophilic agar plates containing in grams per litre: 10 g casein acid hydrolysate, 10 g yeast extract, 5 g proteose peptone, 3 g tri-sodium citrate, 2 g potassium chloride, 25 g magnesium sulphate and 20 g agar supplemented with 10% NaCl adjusted to pH 7.2 ± 0.2 (at 25 °C) and incubated at 35 °C for 5 days.

### Morphological characterization

Morphological characterization of all isolates were studied by plating on halophilic agar plates and incubating at 35 ± 2 °C for 24 h in BOD incubator. The morphological and cultural characteristics such as size, shape, elevation, margin, texture, opacity and pigment with regard to colonial characters were then observed and recorded from the growth on halophilic agar plates. In addition it also included microscopic characterization by Gram’s staining, motility and scanning electron microscopic observation.

### Determination of salt, pH and temperature tolerance test

The salt, pH and temperature tolerance of all isolates were tested separately in halophilic broth tubes supplemented with NaCl concentrations ranging from 5, 10, 15, 20 and 25 per cent for salt tolerance, pH ranging from 2.0, 4.0, and 6.0, 8.0 and 10.0 for pH tolerance, and the temperature ranging from 18, 25, 35 and 45 °C for temperature tolerance. These tubes were inoculated with 0.1 ml (having 10^7^ CFU ml^−1^) previously grown culture of all isolates. A tube without inoculation served as the negative control for each range of salt, pH and temperature respectively. The isolates were inoculated and incubated at 35 ± 2 °C for 8–10 days. The strain that could grow at particular range of salt, pH and temperature were considered as tolerant by observing the presence or absence of growth and comparing it with negative control respectively for each isolates.

### Biochemical characterization

Biochemical characterization of isolates were carried out by using a HiPure Bacterial Identification Kit obtained from HiMedia for biochemical tests viz*.,* glucose, adonitol, lactose, arabinose, sorbitol, citrate, lysine and ornithine utilization, urease detection, phenyl alanine deamination, nitrate reduction and H_2_S production tests. Other biochemical tests viz*.,* starch, lipid and gelatin hydrolysis, indole production, methyl red, Voges–Proskauer and catalase tests were carried out manually according to the standard protocol followed by Nezami et al*.*^45^ while oxidase test was based on the method described by Nyakeri^46^.

### IAA production

In vitro IAA production of isolates were determined following the protocol described by Khalid et al*.*^47^ on a freshly prepared 10 ml Glucose Phosphate Broth (GPB) medium prepared in 100 ml Erlenmeyer flasks. l-Tryptophan was added at desired concentration to the liquid medium by filter sterilization and passing through 0.2 μm membrane filter. 1.0 ml of 3-days old isolates broth (10^7^ CFU ml^−1^) were then inoculated into each flasks and incubated at 30 ± 2 °C for 48 h. A flask containing the same broth medium without inoculation was kept as negative control for comparison. Following incubation, the isolates’ broth culture contents were filtered through Whattman filter paper No.2 and 3.0 ml of culture filtrate were taken in test tubes and 2.0 ml of Salkowski’s reagent was added for measuring IAA production. The test tubes containing the contents were then allowed to stand for 1/2 h for color development. The colour development were observed in both standard solutions and culture filtrate of IAA and the intensity of color developed were measured by spectrophotometer at 530 nm and recorded. The concentration of IAA produced by the bacterial isolates were then deduced by calculation from the IAA standard curve.

### Nitrogen fixation capacity

The nitrogen fixing capacity of the selected halophilic bacterial isolates were studied on a nitrogen free Jensen’s agar medium. The plates were inoculated by streaking the isolate culture onto the plates and incubated at 35 ± 2 °C for 5 days. The nitrogen fixation capacity of the bacterial isolates were then determined by micro Kjeldahl digestion and distillation method^48^. The uninoculated plate of the same media was used as negative control. The nitrogen content in the nitrogen free media which is equivalent to the amount of nitrogen fixed by the isolates were then determined by calculating with the following formula:

$$\begin{aligned} {\text{Nitrogen content }} ( \% ) &= [ ( {\text{ml of }}0.05{\text{ N sulphuric acid for sample }} - {\text{ ml of }}0.05{\text{ N sulphuric}} \\ &\quad {\text{acid for blank}} ) \times 0.05 \times 0.014 \times 100 ]/{\text{Mass of sample }} ({\text{g}})] \end{aligned}$$

where, 0.05 = Normal concentration of H_2_SO_4_ used; 0.014 = Conversion factor.

### Qualitative phosphate solubilization test in solid medium

Qualitative phosphate solubilization test in Sperber’s medium was carried out as per standard protocol followed by Nosrati et al*.*^49^. To examine Pi (Inorganic phosphorus) solubilization capabilities, 10 μl of the bacterial suspensions (~ 10^4^ CFU ml^−1^) was spotted with the help of sterile inoculating needle onto the center of Sperber medium plate containing insoluble Pi. The inoculated plates were incubated at 28 °C. A Sperber agar plate spotted with *E. coli* was kept as the negative control. The zone of solubilization was recorded at 3, 5, 7 and 10 days after inoculation. The Solubilization Index (SI) was determined by measuring the ratio of halo (clear zone) diameter (mm) and the colony diameter as per the formula followed by Nautiyal^50^. And the Solubilization Efficiency (SE) was calculated by the formula followed by Nguyen et al*.*^51^.

$$\begin{aligned} {\text{SI }} &= ( {{\text{Colony diameter }} + {\text{ diameter of halo}}} )/{\text{Colony diameter}}\\ {\text{SE }}( \% ) &= ({\text{Solubilization diameter }}/{\text{Colony diameter}}) \times 100 \end{aligned}$$

### Quantitative phosphate solubilization test in liquid medium

Quantitative phosphate solubilization test in liquid medium was carried out in phosphate solubilization medium as per standard protocol followed by Dahale^52^. Erlenmeyer flasks (250 ml) containing 50 ml of phosphate solubilization estimation medium (containing per litre: 0.5 g yeast extract, 10 g dextrose, 5 g CaCl_2_, 0.5 g (NH_4_)_2_SO_4_, 5 g Ca_3_(PO_4_)_2_, 0.2 g KCl, 0.1 g MgSO_4_, 0.0001 g MnSO_4_ and 0.0001 g FeSO_4_, pH 7.0) were inoculated with 100 μl of bacterial suspension (approx. 10^7^ CFU/ml) in triplicates and incubated on rotary shaker (180 rpm) at 28 °C. After an intervals of 3, 5, 7 and 10 days, samples were drawn aseptically and centrifuged at 5000×*g* for 10 min to pellet the cell biomass and insoluble phosphate and the supernatants were used for the measurement of pH using pH meter for the determination of acidity and liberated Pi following phosphomolybdic blue color method^42^. The amount of Pi released in respective broths were estimated from three flasks each after incubation (DAI) in comparison with a set of uninoculated control. The concentration of Pi released were then calculated by plotting a graph of OD versus concentration of phosphates released in μg for standard and samples followed by their comparisons.

### Estimation of pH change in broth culture

The supernatant obtained after centrifugation of each isolate culture at 3, 5, 7 and 10 DAI were examined for change in pH for determining acidity using a digital pH meter. The drop in pH in supernatants of each respective culture broth were recorded by comparing with the initial pH kept at 7.0.

### Estimation of Pi released from culture supernatant

The available phosphorous content in the broth supernatant of each respective isolates were estimated by following phosphomolybdic blue color method^42^. A known varying concentration of potassium dihydrogen phosphate was used to prepare the standard curve. One ml supernatant of each isolates and control were then taken in 50 ml volumetric flask and 10 ml of chloromolybdic acid was added to it and the contents were mixed thoroughly. The volume was adjusted to three fourth with distilled water and 0.25 ml chlorostannous acid was added followed by immediate adjustment of volume to 50 ml with distilled water. After 15 min, the intensity of blue color developed were measured in UV spectrophotometer at 610 nm using reagent blank.

### Potash solubilization capacity

The potash solubilization capacity of the selected halophilic bacterial isolates were tested qualitatively on an aleksandrow agar plates. A loopful of bacterial cells were picked from the respective fresh isolate culture broth and spotted in the middle of the plates containing solidified medium. The inoculated plates were then incubated in the incubator by placing them upside down at 30 °C for 4 days. An Aleksandrow agar plate spotted with an *E. coli* culture in the centre served as the negative control. The different bacterial isolates showing ability to solubilize potash by forming zone around their colony growth were considered as positive potash solubilizing bacteria. The diameter of zone of clearance or solubilization (halo) observed around the bacterial colony and the diameter of colony were measured after 3, 5, 7 and 10 days of inoculation in triplicates. Potash solubilization index (KSI) was then calculated as the ratio of diameter of halo (mm)/diameter of colony (mm) as per the formula followed by Nautiyal^50^. And the Solubilization Efficiency (SE) was calculated by the formula followed by Nguyen et al*.*^51^.

### Siderophore production potentiality

The siderophore production potentiality of isolates were determined by following qualitative plate assay on Chrome Azurol S blue agar medium (CAS) to detect the siderophore production by isolates as per method described by Bhatt^53^. The CAS agar media was prepared according to the step-by-step procedure described by Louden et al*.*^54^. The individual CAS plates were then spot inoculated by overnight grown cultures of respective isolates and incubated at 30 ± 2 °C for 24 h. The isolates showing yellow to orange colored ring around the colonies were then considered as positive siderophore producing strains. The siderophore types produced were further identified as hydroxymate, catechol or carboxylate type by Tetrazolium, Arnow’s, and Carboxylate test respectively as per the standard protocol described by Bhatt^53^.

### Qualitative ACC deaminase production

Qualitative ACC deaminase production test was carried out using a simple plate assay following the standard protocol^38^ in Dworkin and Foster (DF)^55^ minimal salts medium containing per litre of distilled water: 4.0 g KH_2_PO_4_, 6.0 g Na_2_HPO_4_, 0.2 g MgSO_4_.7H_2_O, 2.0 g glucose, 2.0 g gluconic acid and 2.0 g citric acid with trace elements: 1 mg FeSO_4_.7H_2_O, 10 μg H_3_BO_3_, 11.19 μg MnSO_4_.H_2_O, 124.6 μg ZnSO_4_.7H_2_O, 78.22 μg CuSO_4_.5H_2_O, 10 μg MoO_3_, adjusted to pH 7 and then supplemented with 1.8% (w/v) Bacto-Agar (Difco Laboratories, Detroit, MI, USA) which has a very low nitrogen content. After sterilizing the media by autoclaving at 15 psi 121 °C for 20 min, the heat-labile ACC was filter-sterilized through a 0.2-mm membrane at a concentration of 3 mM and the filtrate was added to the DF salts minimal medium as a sole nitrogen source and poured onto each plates marked as DF-ACC. After solidification, the plates were spot inoculated with fresh isolates inoculum and incubated for 72 h at 32 ± 2 °C but not higher than 35 °C to avoid inhibition of ACC deaminase activity of the isolates. Plates containing only DF minimal salts medium without ACC marked as DF plates were used as the negative control and those with (NH_4_)_2_SO_4_ (0.2% w/v) in place of ACC marked as ACC-NH_3_ plates were used as the positive control^56^. Isolates showing growth on ACC-supplemented plates were compared to the negative and positive controls and considered as positive for ACC deaminase production as indicated by their ability to break down ACC and utilize it as the nitrogen source for growth.

### Quantitative ACC deaminase production

The ACC deaminase activity of isolates were estimated by performing quantitative test as described hereafter. The bacterial isolates were cultured first in rich medium and then transferred to minimal medium with ACC as the sole nitrogen source in order to create culture conditions that favors the induction of isolates ACC deaminase activity. The isolates exhibiting positive ACC deaminase activity in the qualitative test were cultured first in a 15 ml fresh TSB broth and incubated at 35 ± 2 °C for 24–48 h on a shaker incubator at 150–200 rpm. After incubation, the accumulated biomass of the respective isolate cultures were harvested by centrifugation at 8000 rpm for 10 min at 4 °C and supernatants were discarded. The cell pellet of each isolates were then washed with 5 ml of either 0.1 M Tris–HCl (pH 7.6) or fresh DF salts minimal medium and centrifuged again and pellets resuspended in 15 ml DF minimal salt medium containing ACC at a final concentration of 3 mM in triplicates and incubated at 32 °C for another 36–72 h on a shaker incubator. The bacterial cells were then harvested again by centrifugation at 8000 rpm for 10 min at 4 °C and the supernatants were discarded and the cell pellet of each isolates were washed twice with 5 ml of 0.1 M Tris–HCl (pH 7.6) to ensure the pellets were free of the bacterial growth medium^38^ and re-suspended in 1 ml of 0.1 M Tris–HCl (pH 7.6) in new 1.5 ml microcentrifuge tubes. The contents of the 1.5 ml microcentrifuge tubes were centrifuged at 16,000×*g* for 5 min and the supernatants were removed and the pellets were re-suspended in 600 μl of 0.1 M Tris–HCl (pH 8.5). Thirty microliter of 5% toluene (v/v) were added to the respective cell suspensions and vortexed for 30 s to labilize the cells. Then, 200 μl of labilized cell suspensions were transferred to a clean 1.5 ml microcentrifuge tubes and 20 μl of 0.5 M ACC was added and briefly vortexed for 5 s and then incubated at 30 °C for 15 min. About 200 μl of labilized cell suspension without ACC was kept as negative control and 0.1 M Tris–HCl (pH 8.5) with 20 μl of 0.5 M ACC was kept as blank. The samples were then mixed thoroughly with 1 ml of 0.56 N HCl by vortexing and the cell debris removed by centrifugation at 12,000 rpm for 5 min at room temperature and 1 ml of supernatants were transferred to a glass test tube and mixed with 800 μl of 0.56 N HCl and 300 μl of DNP solution i.e. 2,4-dinitrophenylhydrazine reagent (0.2% 2,4-dinitrophenylhydrazine in 2 M HCl). The content mixture were then vortexed and incubated at 30 °C for 30 min. Then 2 ml of 2 N NaOH were added to the sample mixture prepared above and the absorbance were measured at 540 nm in spectrophotometer^57^. Finally, the ACC deaminase activity of isolates were determined by measuring the amount of α-ketobutyrate formed by the cleavage of ACC by ACC deaminase in each samples by comparing their absorbance at 540 nm to a standard curve generated by α-ketobutyrate.

### Molecular identification by 16S rRNA gene amplification

The DNA for 16S rRNA gene amplification were isolated using Qiagen’s DNA extraction DNeasy^®^ Blood and Tissue kit (250) and the 16S rRNA gene amplification were carried out on capillary sequencer (Applied Bio Systems 3130) with the help of a pair of universal oligonucleotide primers viz*.,* forward and reverse primers designated as 005F and 907R with sequences 5′-TGGAGAGTTTGATCCTGGCTCAG-3′ and 5′-CCGTCAATTCMTTTRAGTTT-3′ respectively. The partial 16S rRNA gene sequence obtained from studied bacteria were analyzed and identified with nucleotide BLAST search in Gene Bank of NCBI.

### Consent to participate/publish

I, as the main corresponding author of this paper hereby state that the entire research work was carried out with the appropriate consent of all the concerned authors and that all the concerned authors in relation to this work described has given their approval for participation into this publication process.

## Conclusions

From the present study it was concluded that the halophilic and halotolerant bacterial isolates from agricultural soils of coastal regions of Saurashtra, Gujarat exhibited a very good plant growth promoting characteristics and represent a potent source of biofertilizers as plant growth promoting halobacteria and may help contribute to sustainable agriculture in such adverse conditions through reclamation and recovery of saline soils by supporting crop growth and metabolisms. However, the reported activities of the isolated halotolerant and halophilic bacteria represent only a potential for plant growth promotion. Further inoculation with the candidate PGP-bacteria and salt stressed plants in salt affected soil are necessary for a more complete assessment of plant growth promotion abilities.

## Supplementary Information

Supplementary Information.

Supplementary Figures.

## Data Availability

The main author hereby declare with the consent of all concerned co-authors that data and materials related with the work described would only be made available at request.
