# Supplementary material for: Plant growth promoting characteristics of halophilic and halotolerant bacteria isolated from coastal regions of Saurashtra Gujarat
Source: Sci Rep. 2022 Mar 18;12:4699. doi: 10.1038/s41598-022-08151-x (PMC8933404; doi:10.1038/s41598-022-08151-x)
Supplement: Supplementary file 1 — Supplementary Information. [file 41598_2022_8151_MOESM1_ESM.pdf]

16S rRNA partial gene sequence of two strains submitted to NCBI, GeneBank for the first time from Saurashtra, Gujarat, India

Accession number **MK955347** and **MK961217**

Halomonas pacifica strain HPSB1 16S ribosomal RNA gene, partial sequence

GenBank: MK955347.1

FASTA Graphics

Go to:

```
LOCUS       MK955347                 826 bp    DNA     linear   BCT 27-MAY-2019
DEFINITION  Halomonas pacifica strain HPSB1 16S ribosomal RNA gene, partial
            sequence.
ACCESSION   MK955347
VERSION     MK955347.1
KEYWORDS    .
SOURCE      Halomonas pacifica
  ORGANISM  Halomonas pacifica
            Bacteria; Proteobacteria; Gammaproteobacteria; Oceanospirillales;
            Halomonadaceae; Halomonas.
REFERENCE   1  (bases 1 to 826)
  AUTHORS   Reang,L., Bhatt,S.B., Tomar,R.S., Vyas,U., Joshi,K., Padhiyar,S.,
            Desai,H. and Kheni,J.V.
  TITLE     Halomonas pacifica strain HPSB1 16S ribosomal RNA gene, partial
            sequence
  JOURNAL   Unpublished
REFERENCE   2  (bases 1 to 826)
  AUTHORS   Reang,L., Bhatt,S.B., Tomar,R.S., Vyas,U., Joshi,K., Padhiyar,S.,
            Desai,H. and Kheni,J.V.
  TITLE     Direct Submission
  JOURNAL   Submitted (22-MAY-2019) Department of Biotechnology, Junagadh
            Agricultural University, Motibaugh, Junagadh, Gujarat 362001, India
COMMENT     ##Assembly-Data-START##
            Sequencing Technology :: Sanger dideoxy sequencing
            ##Assembly-Data-END##
FEATURES             Location/Qualifiers
     source          1..826
                     /organism="Halomonas pacifica"
                     /mol_type="genomic DNA"
                     /strain="HPSB1"
                     /isolation_source="Coastal Region of Saurashtra"
                     /db_xref="taxon:77098"
                     /country="India"
     rRNA            <1..>826
                     /product="16S ribosomal RNA"
ORIGIN
        1 gagccgaaac gatggaagct tgcttccagg cgtcgagcgg cggacgggtg agtaatgcat
       61 aggaatctgc ccgatagtgg gcgataacct gcggaaactc aggctaatac cgcatacgtc
      121 ctacgggaga aagcagggga tcttcggacc ttgcgctatc ggatgagcct atgtcggatt
      181 agctagttgg tgaggtaacg gctcaccaag gcgacgatcc gtagctggtc tgagaggatg
      241 atcagccaca ctgggactga gacacggccc agactcctac gggaggcagc agtggggaat
      301 attggacaat gggggaaacc ctgatccagc catgccgcgt gtgtgaagaa ggccttcggg
      361 ttgtaaagca ctttcagcga ggaagaaggc ctgagggcta ataccettca ggaaggacat
      421 cactcgcaga agaagcaccg gctaactccg tgccagcagc cgcggtaata cggagggtgc
      481 gagcgttaat cggaattact gggcgtaaag cgcgcgtagg tggcttgata agccggttgt
      541 gaaagccccg ggctcaacct gggaacggca tccggaactg tcaggctaga gtgcaggaga
      601 ggaaggtaga attcccggtg tagcggtgaa atgcgtagag atcgggagga ataccagtgg
      661 cgaaggcggc cttctggact gacactgaca ctgaggtgcg aaagcgtggg tagcaaacag
      721 gattagatac cctggtagtc cacgccgtaa acgatgtcga ctagccgttg ggtccttga
      781 gacctttgtg gcgcagttaa cgcgataagt cgaccgccct gggata
```

Halomonas stenophila strain HPSB2 16S ribosomal RNA gene, partial sequence

GenBank: MK961217.1

FASTA Graphics

Go to:

```
LOCUS       MK961217                 709 bp    DNA     linear   BCT 28-MAY-2019
DEFINITION  Halomonas stenophila strain HPSB2 16S ribosomal RNA gene, partial
            sequence.
ACCESSION   MK961217
VERSION     MK961217.1
KEYWORDS    .
SOURCE      Halomonas stenophila
  ORGANISM  Halomonas stenophila
            Bacteria; Proteobacteria; Gammaproteobacteria; Oceanospirillales;
            Halomonadaceae; Halomonas.
REFERENCE   1  (bases 1 to 709)
  AUTHORS   Reang,L., Bhatt,S., Tomar,R., Vyas,U., Joshi,K., Padhiyar,S.,
            Desai,H. and Kheni,J.V.
  TITLE     Isolation, characterization and identification of halophilic
            bacteria from the soils of coastal regions of Saurashtra
  JOURNAL   Unpublished
REFERENCE   2  (bases 1 to 709)
  AUTHORS   Reang,L., Bhatt,S., Tomar,R., Vyas,U., Joshi,K., Padhiyar,S.,
            Desai,H. and Kheni,J.V.
  TITLE     Direct Submission
  JOURNAL   Submitted (23-MAY-2019) Department of Biotechnology, Junagadh
            Agricultural University, Motibaugh, Junagadh, Gujarat 362001, India
COMMENT     ##Assembly-Data-START##
            Sequencing Technology :: Sanger dideoxy sequencing
            ##Assembly-Data-END##
FEATURES             Location/Qualifiers
     source          1..709
                     /organism="Halomonas stenophila"
                     /mol_type="genomic DNA"
                     /strain="HPSB2"
                     /isolation_source="Coastal Region of Saurashtra"
                     /host="India"
                     /db_xref="taxon:795312"
     rRNA            <1..>709
                     /product="16S ribosomal RNA"
ORIGIN
        1 gccaacgatg tcgctagccg ttggggtcct tgagaccttt gtggcgcagt taacgcgata
       61 agtcgaccgc ctggggagta cggccgcaag gttaaaactc aaatgaattg acgggggccc
      121 gcacaagcgg tggagcatgt ggtttaattc gatgcaacgc gaagaacctt acctaccctt
      181 gacatcgtgc gaactttcca gagatggatg ggtgccttcg ggaacgcaca gacaggtgct
      241 gcatggctgt cgtcagctcg tgttgtgaaa tgttgggtta agtcccgtaa cgagcgcaac
      301 ccttgtccct atttgccagc gattcggtcg ggaactctag ggagactgcc ggtgacaaac
      361 cggaggaagg tggggacgac gtcaagtcat catggccctt acgggtaggg ctacacacgt
      421 gctacaatgg tcggtacaaa gggttgcaat gccgcgaggt ggagctaatc ccataaagcc
      481 ggtctcagtc cggatcggag tctgcaactc gactccgtga agtcggaatc gctagtaatc
      541 gtgaatcaga atgtcacggt gaatacgttc ccgggccttg tacacaccgc ccgtcacacc
      601 atgggagtgg actgcaccag aagtggttag cttaaccttc gggggagcga tcaccacggt
      661 gtggttcatg actggggtgg aagtcgtaac aaggtaaccg taagttgtt
//
```
