# Supplementary material for: Plant growth promoting characteristics of halophilic and halotolerant bacteria isolated from coastal regions of Saurashtra Gujarat
Source: Sci Rep. 2022 Mar 18;12:4699. doi: 10.1038/s41598-022-08151-x (PMC8933404; doi:10.1038/s41598-022-08151-x)
Supplement: Supplementary file 2 — Supplementary Figures. [file 41598_2022_8151_MOESM2_ESM.pdf]

Plant growth promoting characteristics of halophilic and halotolerant bacteria isolated from coastal regions of Saurashtra Gujarat

Likhindra Reang*[1, 2], Shraddha Bhatt*[2, 3], Rukam Singh Tomar*[2, 4], Kavita Joshi[2, 5], Shital Padhiyar[2, 5], U. M. Vyas[2, 6]

1Main corresponding author, M.Sc. Agriculture, Email: likhindrareang@gmail.com

2Junagadh Agricultural University, Junagadh, Gujarat, India

3Assistant Professor, Livestock Farm Complex, College of Veterinary Science & A.H., Email: sbbhatt@jau.in

4Associate Research Scientist, Main Oilseed Research Station, Email: rukam@jau.in

5Senior Research Fellow, Department of Biotechnology, Email: kjoshi2804@gmail.com

5Senior Research Fellow, Department of Biotechnology, Email: padhiyar.sm@gmail.com

6Assistant Professor, Department of Plant Pathology, Email: baazraz@jau.in

Supplementary Figures

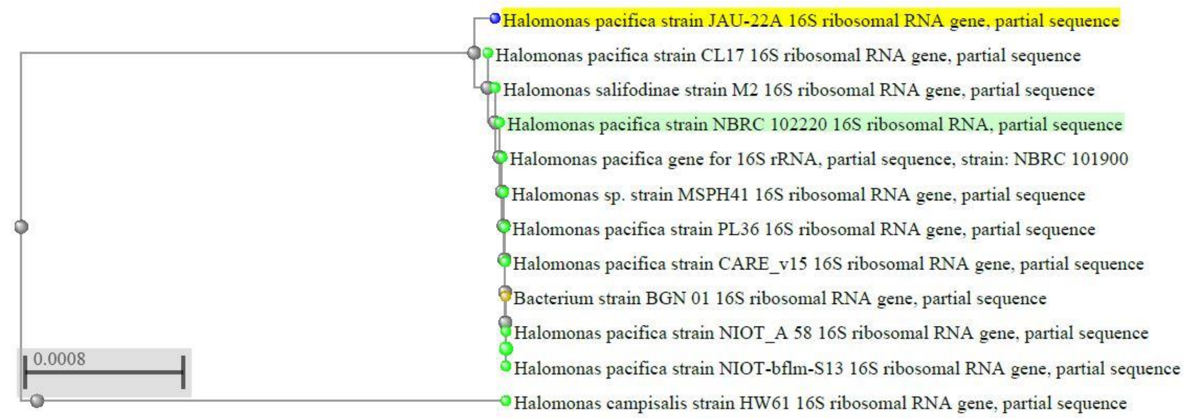

Supplementary Fig. S1. Phylogenetic dendrogram, based on partial 16S rRNA nucleotide sequence, showing the relationship between the halophilic bacterial isolate S_3 designated as strain JAU-22A with closely related taxa of *H. pacifica*.

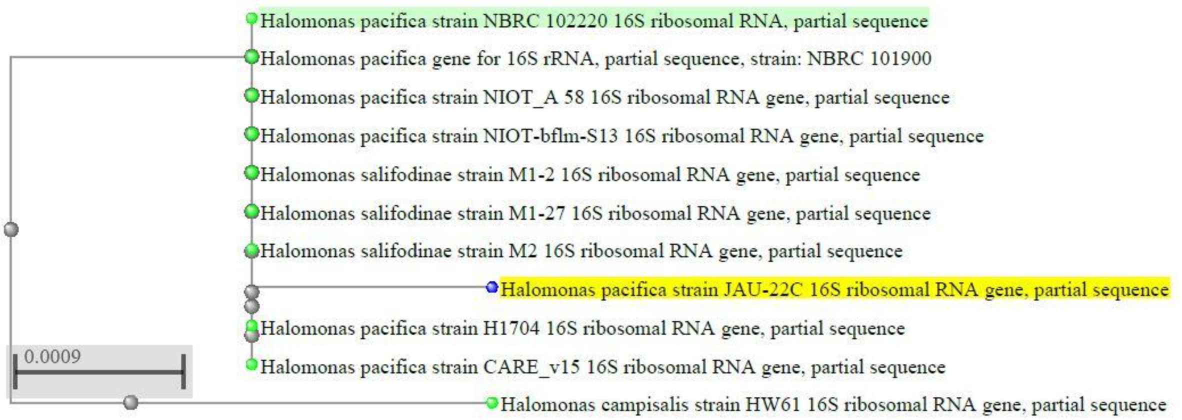

Supplementary Fig. S2. Phylogenetic dendrogram, based on partial 16S rRNA nucleotide sequence, showing the relationship between the halophilic bacterial isolate S_4 designated as strain JAU-22C with closely related taxa of *H. pacifica*.

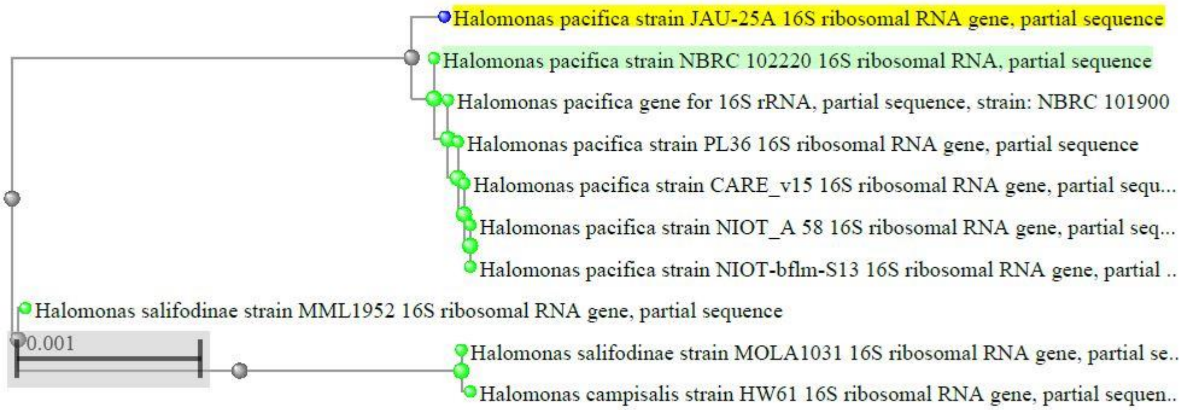

Supplementary Fig. S3. Phylogenetic dendrogram, based on partial 16S rRNA nucleotide sequence, showing the relationship between the halophilic bacterial isolate S_5 designated as strain JAU-25A with closely related taxa of *H. pacifica*.

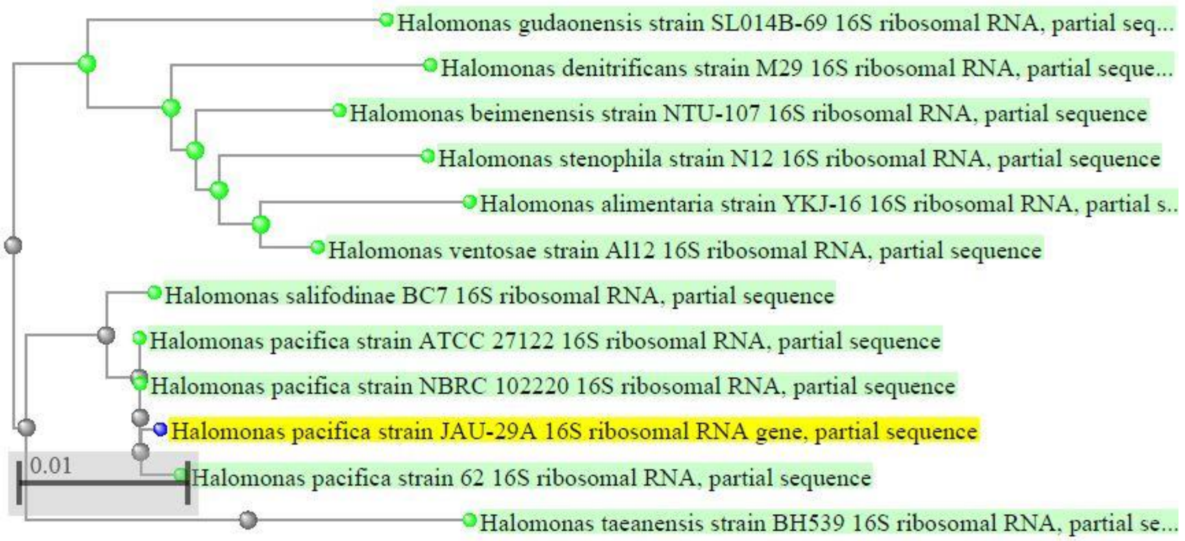

Supplementary Fig. S4. Phylogenetic dendrogram, based on partial 16S rRNA nucleotide sequence, showing the relationship between the halophilic bacterial isolate S_6 designated as strain JAU-29A with closely related taxa of *H. pacifica*.

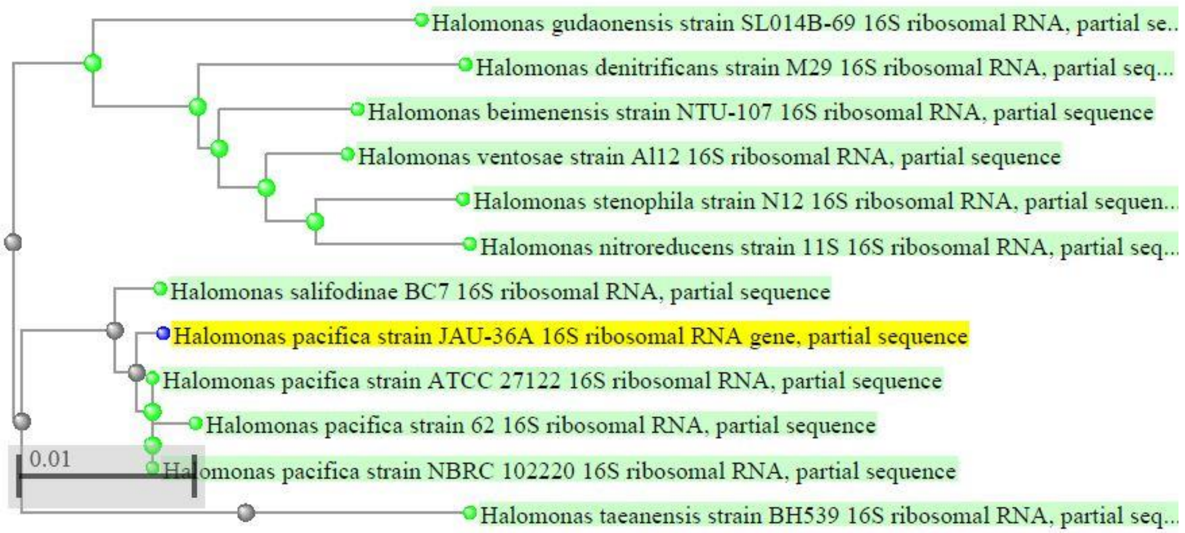

Supplementary Fig. S5. Phylogenetic dendrogram, based on partial 16S rRNA nucleotide sequence, showing the relationship between the halophilic bacterial isolate S_7 designated as strain JAU-36A with closely related taxa of *H. pacifica*.

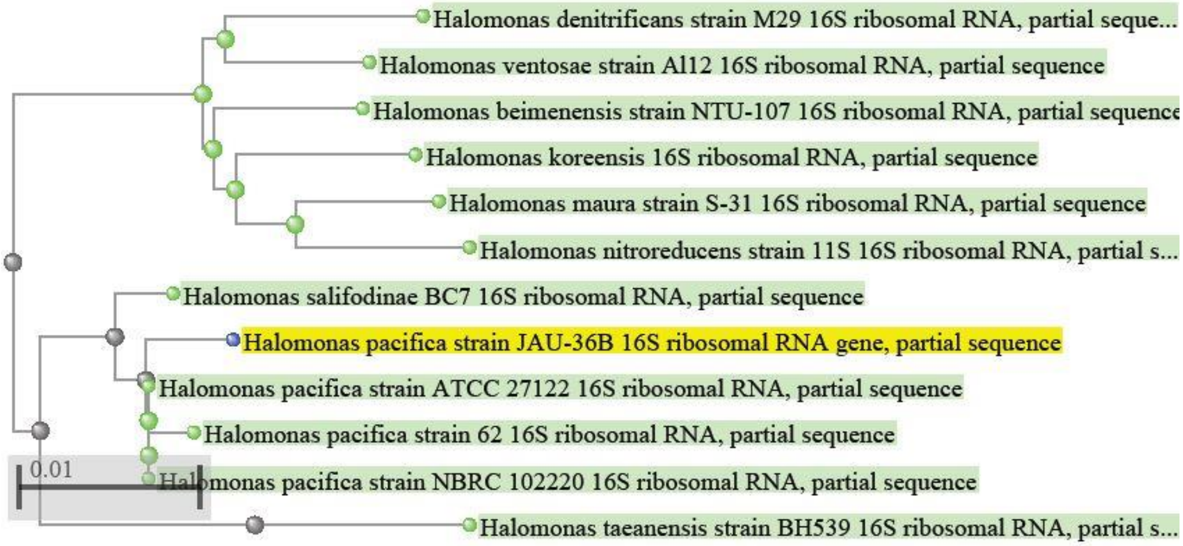

Supplementary Fig. S6. Phylogenetic dendrogram, based on partial 16S rRNA nucleotide sequence, showing the relationship between the halophilic bacterial isolate S_8 designated as strain JAU-36B with closely related taxa of *H. pacifica*.

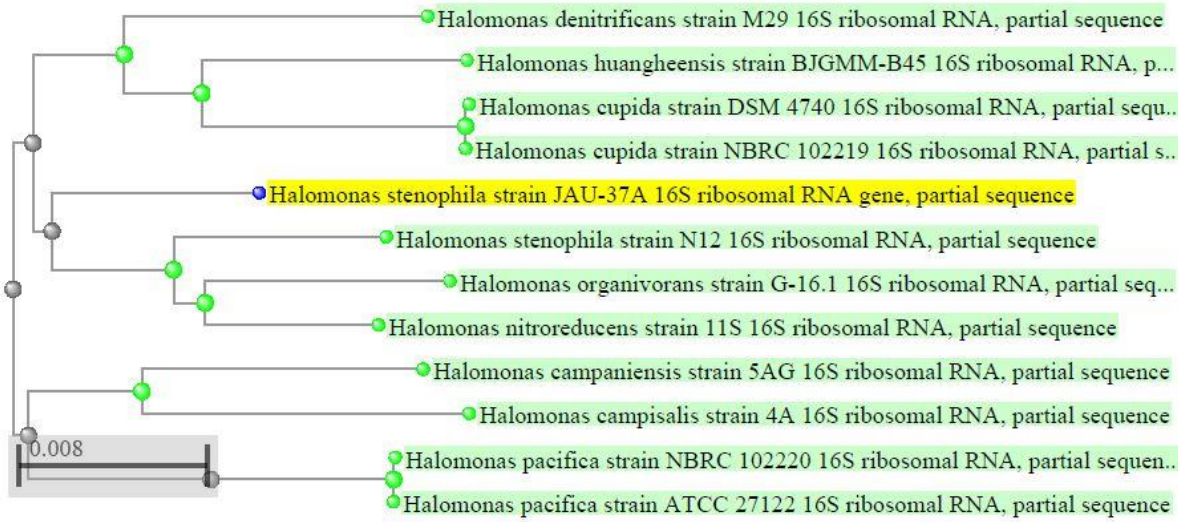

Supplementary Fig. S7. Phylogenetic dendrogram, based on partial 16S rRNA nucleotide sequence, showing the relationship between the halophilic bacterial isolate S_9 designated as strain JAU-37A with closely related taxa of *H. stenophila*.

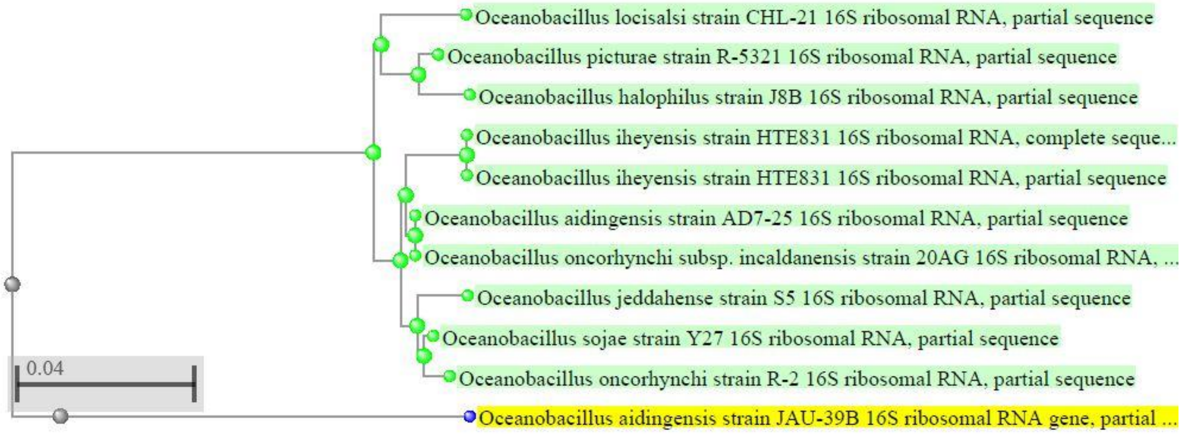

Supplementary Fig. S8. Phylogenetic dendrogram, based on partial 16S rRNA nucleotide sequence, showing the relationship between the halophilic bacterial isolate S_{10} designated as strain JAU-39B with closely related taxa of *O. aidingensis*.

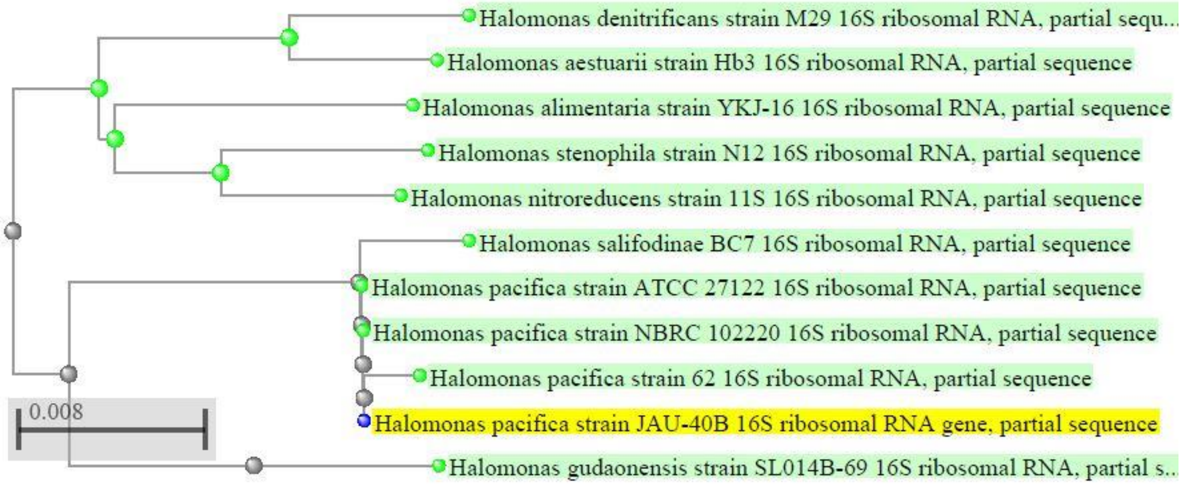

Supplementary Fig. S9. Phylogenetic dendrogram, based on partial 16S rRNA nucleotide sequence, showing the relationship between the halophilic bacterial isolate S_{11} designated as strain JAU-40B with closely related taxa of *H. pacifica*.

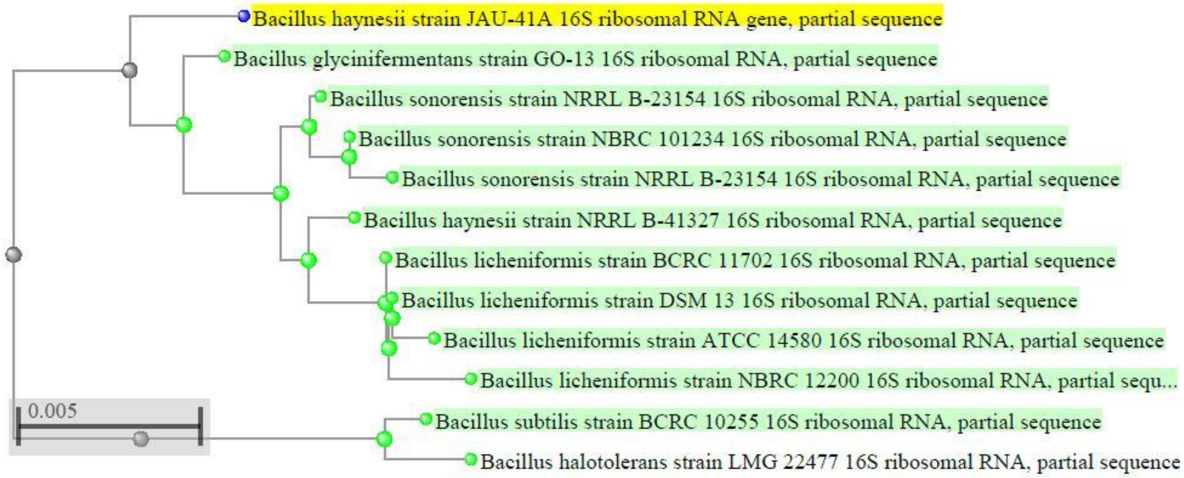

Supplementary Fig. S10. Phylogenetic dendrogram, based on partial 16S rRNA nucleotide sequence, showing the relationship between the halophilic bacterial isolate S_{12} designated as strain JAU-41A with closely related taxa of *B. haynesii*.

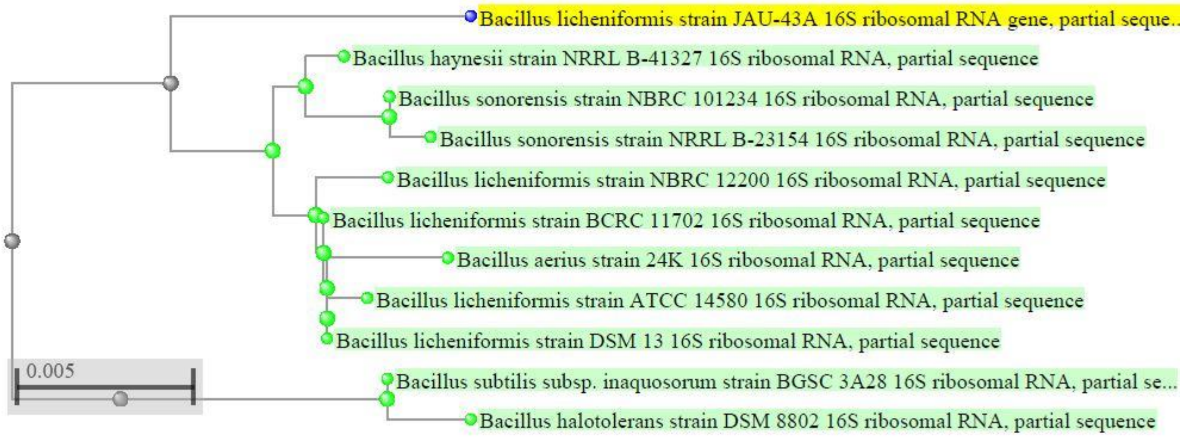

Supplementary Fig. S11. Phylogenetic dendrogram, based on partial 16S rRNA nucleotide sequence, showing the relationship between the halophilic bacterial isolate S_{13} designated as strain JAU-43A with closely related taxa of *B. licheniformis*.

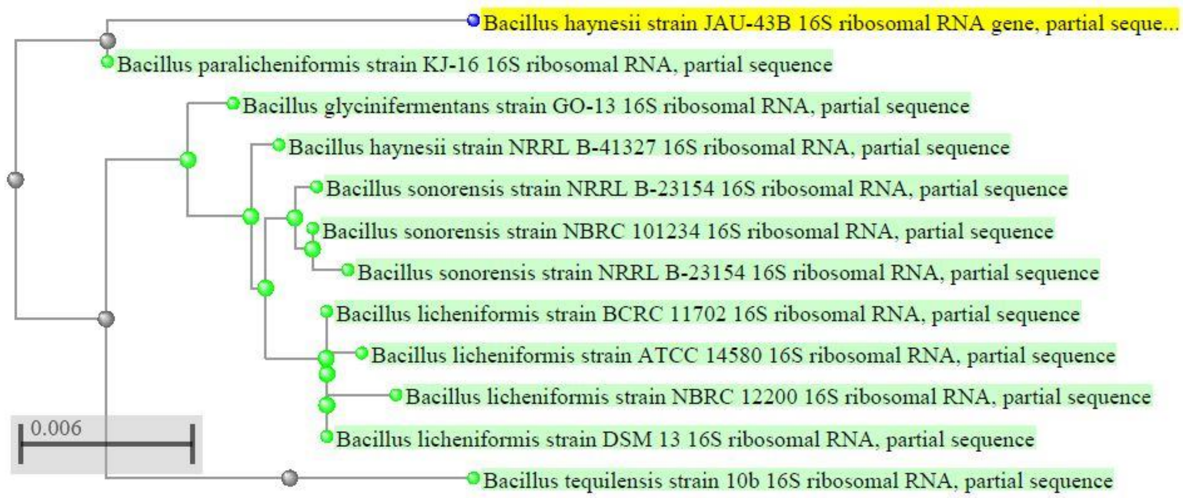

Supplementary Fig. S12. Phylogenetic dendrogram, based on partial 16S rRNA nucleotide sequence, showing the relationship between the halophilic bacterial isolate S14 designated as strain JAU-43B with closely related taxa of *B. haynesii*.

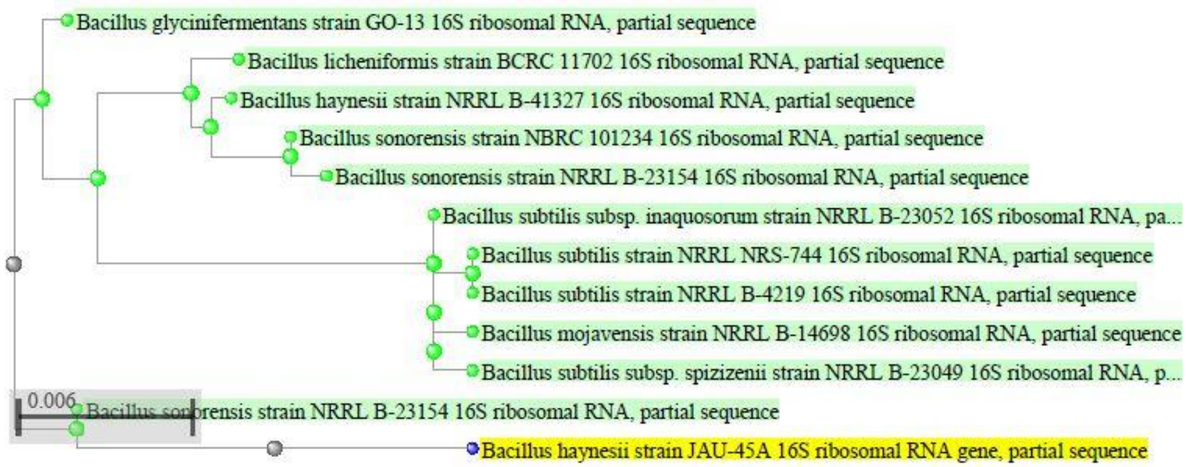

Supplementary Fig. S13. Phylogenetic dendrogram, based on partial 16S rRNA nucleotide sequence, showing the relationship between the halophilic bacterial isolate S15 designated as strain JAU-45A with closely related taxa of *B. haynesii*.
